# Artificial Intelligence Readiness in Clinical Trial Operations: A Narrative Review and Site-Level Governance Framework

**DOI:** 10.3390/healthcare14162519

**Published:** 2026-08-12

**Authors:** Simona Wójcik, Anna Rulkiewicz, Justyna Domienik-Karłowicz

**Affiliations:** 1 LUX MED Sp. z o.o., 02-678 Warsaw, Poland; anna.rulkiewicz@luxmed.pl; 2 University of Medical Sciences, 02-678 Warsaw, Poland; justyna.domienik-karlowicz@luxmed.pl; 3 Department of Internal Medicine and Cardiology, Medical University of Warsaw, 02-005 Warsaw, Poland

**Keywords:** artificial intelligence, clinical trials, clinical trial operations, large language models, patient recruitment, risk-based quality management, Good Clinical Practice, AI governance, site readiness, health informatics

## Abstract

**Background/Objectives:** Artificial intelligence (AI) is increasingly introduced into clinical trial operations, but operational usefulness does not imply regulatory or site-level readiness. This review maps AI applications across trial operations and proposes an author-developed, unvalidated site-level readiness framework and preliminary deployment-decision aid. **Methods:** We conducted a structured narrative review with evidence mapping of peer-reviewed literature, regulatory documents and contextual sources (2020–2026). AI use cases were mapped by lifecycle stage, technical-validity reporting, evidence maturity, autonomy, trial impact and governance implications. Maturity was assessed with an author-developed 0–8 score intended for transparent mapping, not risk-of-bias grading. **Results:** The comparatively strongest evidence concerns patient–trial matching and eligibility assessment, which nonetheless reached only moderate maturity, being evaluated mainly retrospectively or in simulated screening rather than inside a live trial; no use case reached the highest band. Other applications remain less mature or context-dependent. Recurrent risks include hallucination, automation bias, weak local validation, limited auditability, model drift and unclear accountability. We propose a preliminary framework linking evidence maturity, technical validity, AI autonomy, trial impact and site capacity. **Conclusions:** AI readiness in clinical trial operations should be assessed at the level of the AI-enabled workflow rather than the model alone. Safe adoption requires context-specific technical and operational validation, human accountability, auditability, lifecycle monitoring and alignment with Good Clinical Practice.

## 1. Introduction

Clinical trials remain the cornerstone of evidence generation in medicine, yet they are widely regarded as slow, expensive, and operationally fragile. Rising protocol complexity, difficulty in identifying and enrolling eligible participants, growing volumes of heterogeneous data, and the progressive decentralization of trial activities have all increased the operational burden placed on sponsors, contract research organizations and, in particular, investigational sites and the healthcare organizations that host them [1,2]. Site burden is now a measurable phenomenon: survey-based work quantifying protocol-related site burden across hundreds of sites has linked excessive procedural and administrative load to slower performance [3]. Against this background, artificial intelligence (AI)—and especially machine learning (ML) and, more recently, large language models (LLMs) and generative AI—has been proposed as a way to relieve specific bottlenecks in trial design, recruitment, monitoring, safety surveillance and documentation [2,4,5]. These developments build on broader early appraisals of GPT-4 in healthcare, which described its potential role in medical education, triage, documentation and clinical communication while emphasizing persistent limitations and the need for professional oversight [6].

Much of the existing literature frames AI in clinical trials as a collection of promising but isolated applications. However, AI is now moving from the automation of discrete tasks toward workflow-embedded systems that support, augment, or partially automate activities across the entire trial lifecycle [1,4]. This transition changes the relevant questions. When AI performs a one-off calculation, the central issue is accuracy. When AI becomes embedded in operational workflows that influence who is screened, which sites are selected, how deviations are detected and what participants are told, the central issue becomes governance: validation, human oversight, auditability, accountability and alignment with Good Clinical Practice (GCP) [5,7].

In this review, we use the term AI as an operational layer to describe workflow-embedded AI systems that support or partially automate activities across protocol design, feasibility assessment, site selection, recruitment, eligibility assessment, trial conduct, monitoring, safety surveillance, documentation and regulatory oversight. This framing does not imply autonomous replacement of investigators, sponsors or trial staff; rather, it emphasizes that the operational value of AI is inseparable from the conditions under which its outputs are validated, supervised, documented and governed.

The operational rationale for AI adoption is real but contextual rather than evidentiary. Industry analyses indicate persistent pressure on clinical development productivity [8], high costs associated with trial delays—with one estimate placing the mean cost of a delay day in drug development at approximately USD 40,000 [9]—and increasing strain on investigational sites [3,10]. Commercial forecasts predict rapid growth in AI-enabled trial solutions but vary substantially across sources [11,12], and registry analyses indicate that AI-related trials are now globally distributed yet unevenly reported [13,14]. These signals help explain why adoption is accelerating, but they do not establish that AI is ready for routine operational use.

The aim of this narrative review is to synthesize current evidence on AI in clinical trial operations, to identify areas of relative operational maturity, to discuss major risks and failure modes, and to propose a site-level governance and readiness framework aligned with GCP, participant safety, data integrity, human oversight and emerging regulatory expectations. We address five questions: (i) which AI applications show the greatest current operational maturity; (ii) what are the main operational, ethical, data-related and regulatory risks; (iii) how can the level of AI autonomy be linked to governance requirements; (iv) which site-level readiness elements should be assessed before implementation; and (v) how can AI readiness be aligned with GCP, human accountability and evolving regulatory expectations. The scope is deliberately restricted to AI in clinical trial operations rather than AI in medicine at large; we exclude, except where directly relevant, AI in drug discovery, routine clinical diagnosis, imaging, robotic surgery, hospital operations unrelated to trials, and AI as a regulated medical device [15].

### Positioning Relative to Existing Literature

Several recent frameworks and reviews have addressed AI adoption in clinical trials or in healthcare more broadly. Mateen and colleagues proposed a lifecycle-level framework for the effective adoption of AI in clinical trials [16], and You and colleagues proposed a clinical-trials-informed pathway for the real-world implementation and deployment of AI in healthcare organizations [17]. Broader guidance for trustworthy and deployable healthcare AI, such as FUTURE-AI, spans the entire lifecycle from design and validation to regulation, deployment and monitoring [18], and practical frameworks for the appropriate implementation and review of healthcare AI have been proposed [19]. Established reporting guidelines improve transparency when AI is the object of study: SPIRIT-AI and CONSORT-AI extend trial protocol and reporting standards to AI interventions [20,21], DECIDE-AI addresses the early-stage clinical evaluation of AI-driven decision-support systems [22], and TRIPOD + AI updates reporting for AI prediction models [23].

These contributions leave a specific and consequential gap. Existing AI-in-clinical-trials reviews primarily provide use-case taxonomies of where AI may be used, and existing AI reporting guidelines primarily address situations in which AI is the intervention, decision-support system, or prediction model under evaluation. Many AI systems likely to enter clinical trial operations, however, will function as workflow infrastructure rather than study interventions: they may generate prescreening lists, summarize source data, prioritize queries, draft participant-facing explanations or flag safety signals, thereby influencing regulated trial conduct without appearing in the protocol as the object of evaluation. This produces an operational AI governance gap at the level of the investigational site—the need to determine when such tools are mature enough to use, what degree of human oversight is required, what documentation must be retained, and how an inspector could reconstruct an AI-influenced decision. In short, the literature no longer lacks awareness that AI can be used in clinical trials; it lacks a site-level implementation logic for determining when, how, and under what governance conditions AI-enabled workflows should be used. The distinctive contribution of this review is to address that gap with a site-level decision model linking four dimensions rarely considered together—evidence maturity, AI autonomy, trial impact, and site implementation capacity—to inspection-ready, GCP-aligned deployment decisions. Box 1. Key contributions of this review to AI-enabled clinical trial operations. The key contributions of this review are summarized in Box 1.

Box 1 **Key contributions of this review to AI-enabled clinical trial operations.**

**What this review adds**

• It distinguishes AI as a clinical-trial intervention from AI as operational trial infrastructure.

• It maps AI use cases across the trial lifecycle by evidence maturity, autonomy level and trial impact.

• It argues that AI readiness should be assessed at the level of the AI-enabled workflow, not the model alone.

• It introduces inspection-ready AI as a practical standard for regulated trial operations.

• It provides an author-developed, preliminary site-level deployment-decision aid and implementation checklist for healthcare organizations hosting clinical trial sites.

## 2. Materials and Methods

We conducted a structured narrative review with evidence mapping. It is intended as a critical, transparent synthesis rather than a quantitative meta-analysis, and no pooled effect estimates or formal risk-of-bias scoring were performed. PubMed/MEDLINE served as the primary indexed database. It was supplemented by Scopus, Web of Science Core Collection and Google Scholar, which were used for citation chaining and for grey literature, and by targeted retrieval from the websites of the U.S. Food and Drug Administration (FDA), the European Medicines Agency (EMA) and the International Council for Harmonisation (ICH). The search covered literature published between 2020 and 2026, and older sources were retained only where conceptually necessary. The structured database search was executed on 13 July 2026. Full search strings, database yields and the eligibility criteria are reported in Supplementary Table S4.

The search was run at two levels of specificity, and the difference between them is itself informative. A broad query combining AI concepts (artificial intelligence, machine learning, large language model, generative AI) with clinical-trial concepts returned 6877 records in PubMed/MEDLINE. That set proved dominated by studies in which AI is a clinical intervention, a diagnostic model, or an outcome-prediction model, rather than infrastructure used to run a trial. A second query restricted the trial concepts to operational trial-conduct terms (eligibility criteria, trial recruitment, trial matching, risk-based monitoring, protocol deviations, clinical data management) and returned 815 records. This operationally specific set served as the candidate search set from which peer-reviewed evidence was purposively selected.

We included peer-reviewed empirical studies, reviews, regulatory and reporting-guidance documents, legal instruments and implementation-science frameworks that addressed AI applied to the conduct of clinical trials. Records were excluded for the following pre-specified reasons: AI evaluated as a clinical intervention, a diagnostic or a prognostic model rather than as trial infrastructure; AI for drug discovery, molecular design or preclinical research; papers on trial recruitment or eligibility criteria with no AI component; AI applications outside clinical trial operations; trial reports in which machine learning served only as a statistical method for outcome analysis; and editorials, letters or conference abstracts without substantive content. Screening, full-text assessment and final selection were performed by the first author and verified by a second author, and disagreements were resolved by discussion. Two explicitly identified preprints were retained only as emerging evidence and were never used as sole support for a maturity classification. Industry, market and registry sources were used strictly as contextual, non-evidentiary material and are reported separately (Supplementary Table S1).

Sources were classified by evidentiary role: empirical operational evidence; review or synthesis evidence; regulatory, legal or reporting guidance; implementation-science framework; and contextual market or registry evidence. Only the first four categories were used to support evidence-maturity or governance conclusions; market and registry sources were used solely to characterize adoption pressure. A narrative design was chosen because the evidence base spans heterogeneous empirical studies, technical validations, regulatory documents, reporting guidelines, implementation frameworks, and contextual sources that are not amenable to quantitative synthesis but do require conceptual integration.

The operationally specific search returned 815 records, which should be interpreted as a candidate operational search set rather than as a fully screened eligible pool. Because this was a structured narrative review, selection within that set was purposive and relevance-based rather than exhaustive, and this is the principal methodological difference between this review and a systematic review. We did not apply a PRISMA-style, count-based screening flow, and we do not claim to have appraised every eligible record. We report the search strings, the search date, and the yield at each level of specificity so that the coverage of the search and the residual risk of selective sourcing can be judged by the reader. In total, 94 sources formed the evidence corpus for the synthesis: peer-reviewed empirical studies and reviews, regulatory, legal and reporting-guidance documents, contextual industry and registry sources, two explicitly identified preprints, and methodological or implementation-science references. Two further studies are cited in the Discussion as external contrast and adjacent evidence and were not part of the maturity-scored corpus. The identification, selection and classification process is summarized in Supplementary Figure S2.

For each source, we extracted the publication year, article type, AI technology, trial lifecycle stage, operational use case, reported benefits, risks or limitations, validation approach, implementation context and relevance to site-level governance. To make the maturity assessment transparent and comparable, we applied a 0–8 evidence-maturity score summing four criteria, each rated 0–2 (Table 1): evidence type, data realism, workflow integration and governance reporting. Scores mapped to four classes: 0–2, emerging; 3–4, limited; 5–6, moderate; 7–8, high. Component subscores are reported in Supplementary Table S2.

The four criteria were not selected arbitrarily. They correspond to the four questions that determine whether an operational AI result can be acted upon at a trial site: what kind of study produced the result (evidence type); whether it was obtained on data resembling the site’s own (data realism); whether the tool was tested inside a workflow rather than offline (workflow integration); and whether oversight, auditability and validation were reported at all (governance reporting). These dimensions map onto established implementation-outcome constructs, in particular feasibility, fidelity and appropriateness [24], onto the workflow and people dimensions of sociotechnical models of health information technology [25], and onto the applicability and reporting expectations of appraisal tools for AI prediction models [26]. We did not include model accuracy as a criterion, because accuracy is the quantity whose operational sufficiency is in question.

Equal weighting was applied deliberately, and it is a simplification that should be stated plainly. We had no empirical basis for differential weights, and any weighting we imposed would have encoded our own assumptions about which dimension matters most. Equal weights keep the mapping legible and auditable. To test whether the conclusions depend on that choice, we recomputed the ranking under two alternative weightings: one that doubled the weight of evidence type, and one that doubled the weight of workflow integration (Supplementary Table S6). The two most mature use cases retained their rank under both. Under the workflow-weighted variant, data cleaning and patient-facing consent AI rose into the same band as safety surveillance, but no use case reached the high band under any weighting. The substantive conclusions are therefore not an artefact of equal weighting.

Evidence maturity, methodological quality and technical validity were treated as distinct concepts, because a prospective study may still have serious methodological limitations whereas a retrospective study may be rigorous and externally validated. The 0–8 score maps operational maturity rather than grading risk of bias or model performance. Because the corpus included empirical studies, reviews, regulatory guidance, legal instruments and implementation frameworks, no single formal risk-of-bias instrument was applicable across all source types; empirical sources were therefore coded separately for methodological concern and applicability, including study design, external validation, technical-performance reporting, fairness or subgroup assessment, and proximity to operational trial workflows. Where reported, we also extracted task-specific accuracy, sensitivity, specificity, calibration, robustness, error rates, fairness or subgroup results, and external-validation evidence. Technical validity is necessary but not sufficient for operational readiness: it was recorded separately (Supplementary Data S1) and is considered jointly with operational maturity in the deployment recommendations, rather than being folded into the 0–8 score.

Scores were assigned at the use-case level, and they reflect the dominant body of evidence for a use case rather than the single best study within it. In operational terms, each use-case score was set to the maturity band supported by the preponderance of directly relevant evidence across the narrower workflows that make up the use case—the level shared by the majority of its constituent workflows and carrying the largest body of operationally relevant studies—rather than by averaging heterogeneous workflows or by adopting the single highest-scoring workflow. Where constituent workflows fell in different bands, the more conservative (lower) band was retained unless the higher band was supported by prospective or in-workflow evidence. The traceable mapping from individual sources to workflow-level ratings and to the aggregated use-case score is provided in Supplementary Data S1 (sheets ‘Source classification’ and ‘Workflow scoring’). Scoring was performed by the first author and verified by a second author, with disagreements resolved by discussion. The score is an evidence-mapping tool that makes the authors’ interpretation explicit and comparable. It is not a validated instrument and not a formal risk-of-bias assessment, and it should not be read as one. Because most of the underlying evidence comes from retrospective analyses and technical validations rather than from prospective use inside live trials, no use case in this corpus reached the high band; the maximum observed score was 6. The full extraction table, source classification, component subscores, scoring rationale, methodological-concern coding, technical-validity extraction, and sensitivity analyses are provided as Supplementary Data S1. Although this was not a systematic review, the manuscript was structured to address key quality expectations for narrative reviews, including justification of importance, clarity of aims, transparent literature search, appropriate referencing, scientific reasoning and relevant presentation of data [27]. Generative AI tools were used to support language editing, conceptual organization and the formatting of tables and figures; they were not used to generate original data or to perform independent evidence selection, and all AI-assisted content was reviewed and verified by the authors. The principal limitations of the method are those inherent to narrative reviews: the absence of a formal, exhaustive search protocol and quantitative bias assessment, potential selection bias and rapid obsolescence.

## 3. Results

### 3.1. Overview of the Evidence Corpus

The final synthesis included 94 sources spanning empirical AI clinical-trial studies, reviews and scoping reviews, regulatory and reporting-guidance documents, implementation-science and sociotechnical frameworks, and contextual registry or market sources. Empirical evidence was concentrated in recruitment and eligibility assessment, whereas patient-facing AI, digital twins, synthetic controls and several monitoring applications were supported by more heterogeneous or early-stage evidence. Consistent with the source classification described in the Methods, only empirical, review, regulatory and implementation-science sources were used to support the evidence-maturity map and governance conclusions that follow, while market and registry sources were used solely to contextualize adoption pressure. The characteristics of the principal evidentiary sources are summarized in Supplementary Table S5, and the identification and classification process is shown in Supplementary Figure S2.

### 3.2. AI Across the Clinical Trial Lifecycle

**Figure 1:**
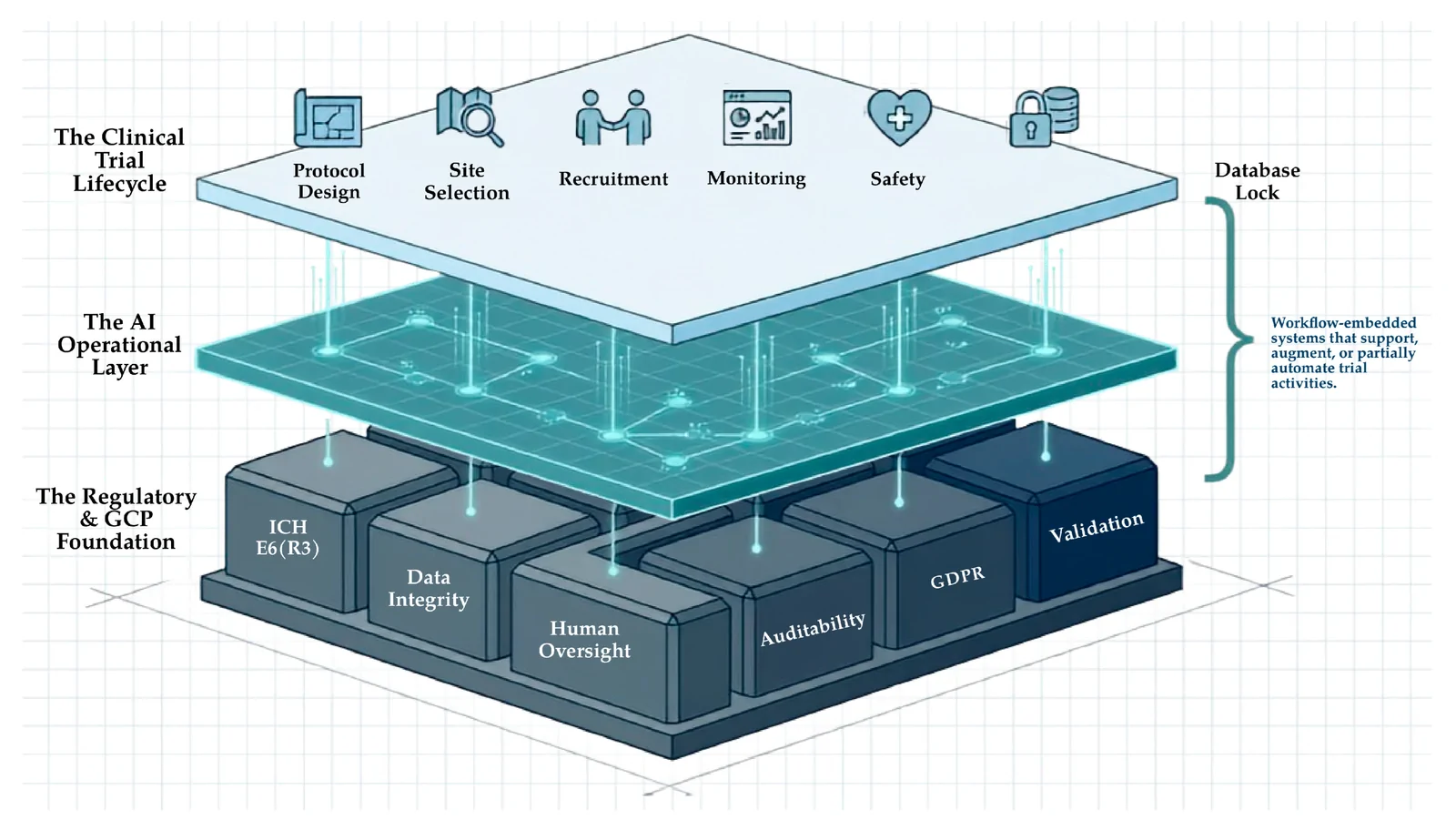
Artificial intelligence as an operational layer in clinical trial operations. The clinical trial lifecycle (top layer) rests on an AI operational layer of workflow-embedded systems that support, augment, or partially automate trial activities, which in turn rests on a foundation of regulatory and Good Clinical Practice requirements (ICH E6(R3), data integrity, human oversight, auditability, data protection, and validation). AI is the connective tissue between lifecycle activities, not the foundation. At each lifecycle stage, the operational layer consumes source or trial data and returns an output that must pass through a documented human checkpoint before it enters the trial record.

AI applications were identified at every stage of the clinical trial lifecycle, from protocol design and feasibility to documentation and regulatory oversight (Figure 1). Rather than a single dominant application, the evidence describes a distributed set of use cases, each with a distinct value proposition, dominant risk, and governance requirement (Table 2). At the design stage, LLMs and information-extraction methods simplify or assess eligibility criteria and other protocol elements [4,28]. In feasibility and site selection, ML models trained on real-world and historical operational data estimate patient pools and rank sites and investigators [29,30,31]. In recruitment and eligibility, LLM-based systems support patient-trial matching and criterion-level screening [32,33,34]. During conduct and monitoring, AI supports risk-based quality management, protocol-deviation detection, and site risk scoring [30,35,36]; in data management, it supports query generation and data cleaning [37,38]. In safety, natural-language methods support adverse-event and adverse-drug-event detection [39,40,41], and in patient-facing processes, generative systems support informed-consent documents and participant education [42,43].

Two observations follow. First, the breadth of applications supports the characterization of AI as an emerging operational layer rather than a peripheral analytical tool. Second, risk is highly stage-dependent: an error in a draft administrative summary is not equivalent to an error in an eligibility decision, a safety signal, or a statement to a participant. A lifecycle map must therefore precede any general claim about readiness.

The operational suitability of AI also depends on the underlying technology. Structured machine-learning models are most relevant to feasibility estimation, site-risk scoring and other operational prediction tasks that rely on tabular real-world data [29,30,31]. Natural-language processing and large language models are central to eligibility-criterion extraction, patient–trial matching, documentation support and consent-related communication [28,32,33,34,42,43]. Generative AI extends these capabilities to summarization, drafting and participant-facing explanation, but it also introduces hallucination and factual-inconsistency risks that make source-grounding, versioning and human verification essential [44,45]. Computer vision is largely confined to imaging-derived endpoints and digital health technologies and therefore sits mostly outside the operational scope considered here, while reinforcement learning remains emerging and is not yet ready for routine operational deployment in trial conduct. The maturity ratings in this review therefore reflect specific technology–task pairings rather than AI in general.

### 3.3. Evidence Maturity of AI Use Cases

**Figure 2:**
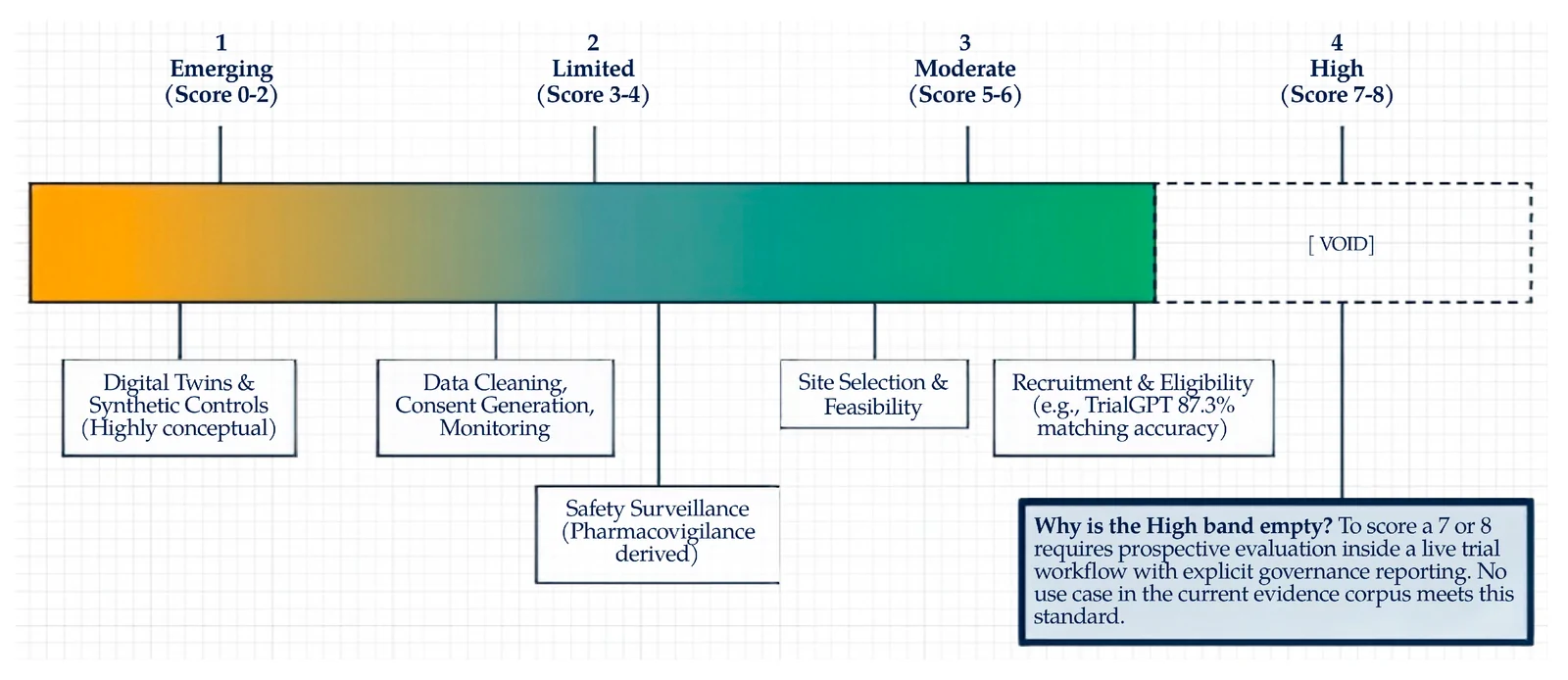
Evidence-maturity of AI use cases in clinical trial operations, positioned along the four maturity bands (emerging, limited, moderate, high). Market and research momentum is concentrated in use cases that have not yet reached the highest band: reaching a score of 7–8 requires prospective evaluation inside a live trial workflow with explicit governance reporting, and no use case in the current evidence corpus meets that standard (high band shown empty). Scores correspond to Table 3.

Applying the scoring rubric (Table 1; component subscores in Supplementary Table S2), operational maturity is uneven (Table 3; Figure 2). The comparatively strongest evidence base concerns recruitment and eligibility assessment, which scored 6 of 8 (moderate), supported by multiple empirical systems, EHR-based validation, and a randomized evaluation of human–AI teaming [28,32,33,34,46]. It is important to be precise about what that rating does and does not mean. It did not reach the high band because the reviewed systems were evaluated in user studies or simulated screening rather than inside a live trial workflow, and governance reporting was partial. No use case in this corpus reached the high band, and the maximum observed score was 6. Site selection and feasibility also scored moderate (5), on the strength of externally validated site-risk models, although the validation is retrospective [29,30,31]. Safety surveillance scored limited (4): the underlying evidence is real-world but comes predominantly from offline pharmacovigilance benchmarks and reviews rather than from trial conduct [39,40,41,47]. Risk-based monitoring, data management and patient-facing consent tools each scored limited (3) [35,36,37,38,42,43,45], and digital twins and synthetic control arms remain emerging (2) [48,49,50]. Therefore, maturity should determine the intended role of AI: cautious pilots with mandatory human review in the more mature areas, and strictly bounded, supervised experimentation or deferral in the less mature ones. A higher maturity rating indicates stronger supporting evidence, not a recommendation to adopt.

### 3.4. Recruitment and Eligibility Assessment as the Leading Use Case

Recruitment and eligibility assessment represent the most operationally mature use case. TrialGPT, an LLM framework combining retrieval, criterion-level matching and ranking, achieved 87.3% accuracy in criterion-level eligibility judgments and reduced screening time by 42.6% in a user study [32]. Whether such gains transfer to routine site workflows has not yet been shown. End-to-end systems such as TrialMatchAI process structured data and clinical narratives and produce explainable, retrieval-augmented outputs suitable for local deployment [33]. PRISM was validated against real-world electronic health record (EHR) data in an oncology center. It showed that a smaller, fine-tuned model can be competitive with larger general-purpose models, which is directly relevant to privacy and local deployment [34]. These systems were, however, developed and evaluated predominantly on English-language clinical records [32,33,34].

**Figure 3:**
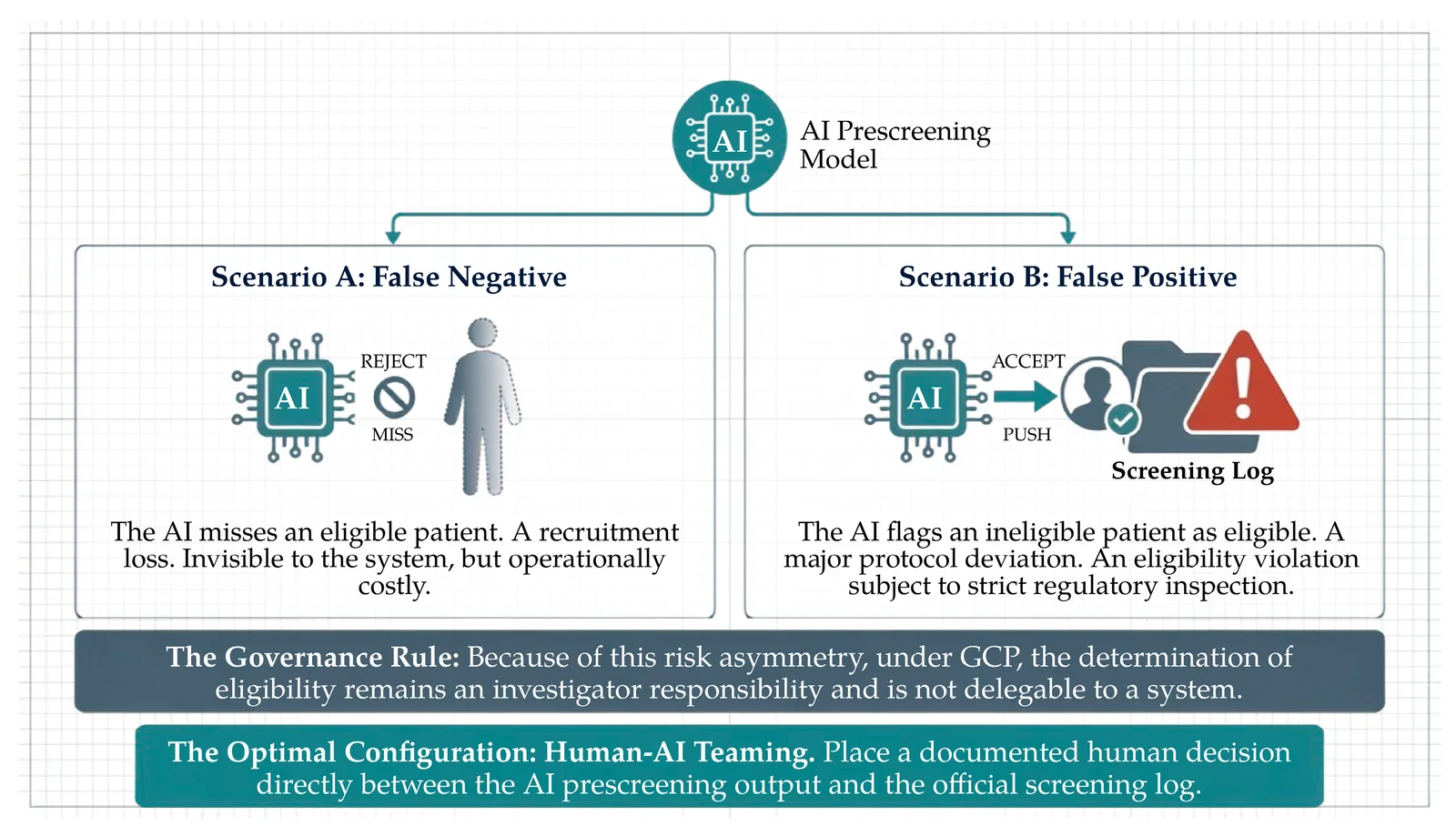
The asymmetry of AI errors in patient recruitment. A false negative (an eligible patient the model does not surface) is a largely invisible recruitment loss; a false positive that leads to enrolment of an ineligible participant is an eligibility violation and a major protocol deviation subject to regulatory inspection. Because the determination of eligibility remains a non-delegable investigator responsibility under Good Clinical Practice, a documented human decision should sit between the AI prescreening output and the official screening log.

Upstream, AutoCriteria extracts granular inclusion and exclusion criteria from protocols [28], while work converting eligibility criteria into standardized database queries shows both the promise of automation and its characteristic failure modes, including hallucination and mapping errors [44]. Broader reviews indicate that recruitment and retention are among the most frequently addressed operational targets for AI [51]. Crucially, the safest configuration is human–AI teaming: a randomized evaluation using retrospective EHR data found that combining AI prescreening with human review improved both accuracy and efficiency relative to either alone [46]. Sociotechnical and economic analyses emphasize that value depends on workflow integration, cost and accountability, not on model accuracy alone [52], and emerging preprint benchmarks should be treated as indicative rather than confirmatory [53]. In practice, recruitment and eligibility tools are best suited to controlled site-level pilots with mandatory human verification rather than to autonomous inclusion or exclusion. At the site level, this often translates into a small extra step: a coordinator confirms a model-prescreened patient against the source record before that patient is entered into the screening log. The two possible errors are not symmetrical. A false negative, meaning an eligible patient the model does not surface, is a recruitment loss and is largely invisible. A false positive that leads to the enrolment of an ineligible participant is an eligibility violation and, in inspection terms, a major protocol deviation. Under GCP, the determination of eligibility remains an investigator responsibility and is not delegable to a system [54]. This asymmetry is the practical reason why a documented human decision should sit between the model output and the screening log (Figure 3).

### 3.5. AI in Trial Conduct, Monitoring and Data Integrity

A scoping review of AI in clinical trial risk assessment identified a substantial literature (142 studies) spanning safety, efficacy and operational risk, but documented fragmentation and limited prospective validation [35]. ML models for site risk prediction have been developed and externally validated for site qualification [30], and methods combining statistical outlier estimation with generative-AI content analysis have been proposed to characterize protocol deviations [36]. In data management, the literature describes a transition toward AI-supported clinical data science, with data cleaning and query generation that may reduce site burden and accelerate database lock [37,38]. These applications illustrate a recurring tension: each efficiency gain raises, rather than lowers, the governance burden—alerts require calibrated thresholds and escalation; AI-assisted data cleaning requires documented human review, traceability and validation; and any model used in oversight must itself be auditable. In practice, AI in monitoring and data management is best positioned as risk-alerting and quality tooling embedded within the existing quality management system rather than as autonomous oversight.

### 3.6. Patient-Facing AI: Consent, Education and Engagement

Patient-facing AI is among the most sensitive applications because it enters directly into communication with participants. LLMs have been evaluated as tools to improve the readability and actionability of informed-consent documents while preserving accuracy [42] and can generate educational materials that support understanding [55]. Conversational systems such as RESPECT show that retrieval-augmented, source-grounded consent assistants can be evaluated for safety and utility, including the capacity to decline out-of-scope questions [43]. Yet evaluations of LLM performance in the consent process reveal a gap: outputs may be highly accurate factually yet fall short on empathy, tone, and contextual sensitivity, so an accurate answer is not necessarily acceptable [45]. Ethical analyses frame AI-supported consent as a balance between improved comprehension and the risks of undue influence and diffusion of responsibility [56]. International guidance on large multi-modal models emphasizes the governance obligations that arise in health communication [57]. Patient-facing AI should also be assessed against its effect on participant trust, comprehension, and willingness to engage [58]. Patient-facing AI should therefore be treated as high-impact communication support requiring bounded, approved and source-grounded content, disclosure, escalation to site staff, and institutional review board and legal review. In a consent conversation, this usually means that an AI system may draft or explain material, while a study nurse or investigator remains the person who answers the participant’s questions and obtains the signature.

### 3.7. Safety Surveillance and Decentralized Data

AI extends into safety surveillance and connects in-trial monitoring with post-marketing signal detection. Reviews describe AI, including LLMs, for adverse-event and adverse-drug-event detection, signal detection and reporting automation, while underscoring limits related to interpretability, data quality and model variability [39,40,41,47]. Because much of this evidence comes from pharmacovigilance and adjacent safety-surveillance contexts, its direct transfer to interventional trial operations should be made cautiously. In parallel, the growing use of digital health technologies and decentralized designs—evidenced by a systematic review of 262 rare-disease trials—generates new, continuous data streams that both enable and complicate AI-supported oversight [59], and regulator alignment on the validation of digital measures offers directly transferable lessons [60]. For this reason, safety-related AI should remain a triage or prioritization layer under investigator and safety-physician review, and the provenance and quality of decentralized data must be assured before such data drive AI-based safety decisions.

### 3.8. Risks and Failure Modes

The principal danger of AI in clinical trial operations is not simply that a model may err, but that its error may go unnoticed, undocumented, or wrongly accepted by a human operator. The reviewed literature converges on a recognizable set of failure modes. Hallucination and mapping errors can produce confident but incorrect outputs—for example, incorrect protocol interpretation or inaccurate participant-facing information [44,45]. Automation bias may lead coordinators or investigators to over-trust recommendations, undermining the human oversight that is meant to make AI safe [46]; in AI-driven clinical decision support, it has been identified as a plausible mechanism of patient harm when users over-rely on imperfect AI outputs [61]. Data bias can systematically disadvantage patients with sparser or lower-quality documentation, distorting eligibility and site-selection decisions [29,34]. Weak local validation means that a model performing well in publication may perform poorly at a given site, while a weak audit trail makes a decision impossible to reconstruct or defend during inspection [37,44]. Finally, model drift degrades performance after deployment, vendor lock-in and cybersecurity exposure introduce external dependencies and privacy risk, and accountability gaps leave it unclear who is responsible for an AI-influenced decision [5,7,50].

One further failure mode falls outside every governed pathway described above, and it may currently be the most common. Unsanctioned use, for example a coordinator pasting an excerpt of a source document into a publicly available chatbot in order to summarize or translate it, leaves no audit trail and is invisible to the sponsor and to monitoring. It also cannot be validated, because the site does not know which system or model version produced the output. Where the text contains participant data, entering it into a public chatbot without a lawful basis, documented instructions and appropriate safeguards is likely to be unlawful processing under Regulation (EU) 2016/679 [62] and may constitute a personal-data breach, depending on the facts; it also departs from GCP expectations for confidentiality and data integrity [54]. Validating sanctioned tools does not address this failure mode at all. It calls instead for an explicit acceptable-use policy, stating which AI systems may be used, with which categories of data, by whom and for which tasks. Such a policy is likely to work better when it is accompanied by a sanctioned alternative for the tasks that staff would otherwise delegate to an unapproved tool. A governance framework that covers only approved AI may therefore cover the smaller part of the problem.

A short vignette makes the failure mode concrete. A coordinator receives a long source-document excerpt and uses a publicly available chatbot to summarize it before completing a screening note. The output is factually plausible but omits an exclusion criterion. The sponsor later detects the mismatch during monitoring, but the site cannot reconstruct which model was used, what text was entered, whether participant identifiers were disclosed, or whether the generated summary influenced the screening log. This is not a failure of a validated AI tool; it is a failure of the surrounding governance environment. The control response should not rely on policy alone. Sites should combine acceptable-use rules with behavioral and technical controls: sanctioned internal or sandboxed AI tools for permitted tasks; role-based access; data-loss-prevention rules; blocking or warning controls for consumer AI services where legally and technically feasible; training based on concrete examples; an easy escalation route for accidental disclosure; and audit logs for approved tools. Governance works best when the compliant path is made easier than the workaround.

### 3.9. AI Autonomy, Trial Impact and Governance Intensity

Risk depends jointly on how much independent action an AI system takes and on how consequential that action is; we therefore treat autonomy and trial impact as two distinct axes and combine them (Table 4). Governance should be proportionate to autonomy, from administrative support through decision support and semi-automated workflows to fully autonomous action. Fully autonomous action is generally not recommended for critical tasks. Consistent with reporting standards for AI decision-support systems, each level should also specify the user role, the human–AI interaction, and explicit override and escalation criteria [22]. Autonomy alone, however, is insufficient: two tools at the same autonomy level may differ greatly in impact, as an internal administrative summary and a participant-facing consent explanation illustrate. Combining the axes yields a simple organizing principle—governance intensity should be determined by the interaction between AI autonomy and trial impact—so that high-impact uses, and any use approaching autonomous action on eligibility, safety or regulatory documentation, demand the most stringent validation, auditability and human accountability.

### 3.10. A Site-Level AI Readiness Framework, Deployment Model and Lifecycle

Sites—and the healthcare organizations that operate them—are not passive users of AI; they are the point at which AI outputs must be verified, documented and translated into accountable trial decisions. The following framework is proposed as a synthesis-derived implementation aid rather than a validated instrument. We propose a site-level readiness framework organized around eleven domains: intended use; data governance; AI-ready data quality; local validation; human oversight; workflow integration; documentation and audit trail; training; vendor qualification; safety escalation; and lifecycle monitoring. Because AI readiness begins with data rather than with the model, we separate general data governance from a distinct AI-ready data-quality domain, reflecting evidence that dataset completeness, standardization and fitness for purpose are prerequisites for reliable AI outputs [63]. For each domain, the framework specifies a site-level question, the responsible role, the required documentation and the risk if the domain is neglected; the minimum documentation a site should assemble is summarized in Box 2, which operationalizes the domains and supports inspection-readiness. In practice, the dossier would be owned jointly by the site or healthcare organization’s research-governance function and the sponsor or contract research organization, with final accountability for trial conduct remaining aligned with GCP-defined sponsor and investigator responsibilities.

Box 2 **Minimum site-level AI implementation dossier (readiness domains operationalized).**

• Intended use: context-of-use and use-case description; risk classification (autonomy level and trial impact)

• Data governance: data-flow map and legal/ethical basis; acceptable-use policy defining permitted AI systems, permitted data categories and permitted users, including prohibition of unsanctioned tools

• AI-ready data quality: data-readiness assessment and quality metrics

• Local validation: validation plan and report, test cases and error analysis; change control and documented release for use

• Human oversight: who signs off; decision log; override and escalation SOP

• Workflow integration: process map and updated standard operating procedure

• Documentation/audit trail: model and version information; source references; versioning; AI use agreed with the sponsor and filed in the investigator site file and trial master file

• Training: training log and competency check; AI-related tasks reflected in the delegation log

• Vendor qualification: vendor and security assessment

• Safety escalation: defined escalation pathway

• Lifecycle monitoring: drift/performance review, incident log, update and retirement criteria

**Figure 4:**
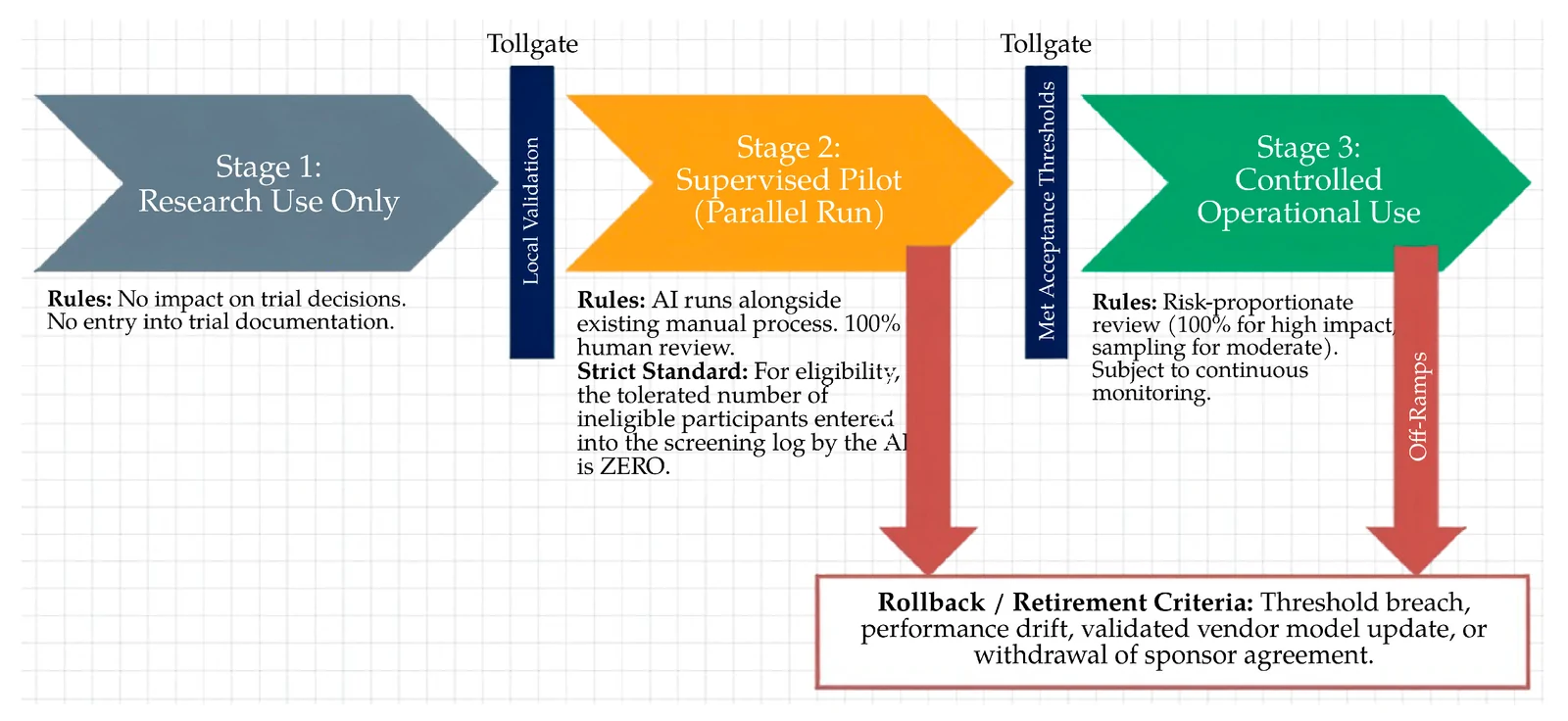
Stage-gated operationalization of an AI-enabled workflow. A use case moves from research use only, through a supervised parallel-run pilot, to controlled operational use, passing a tollgate at each transition (local validation; met acceptance thresholds). Defined off-ramps return the tool to a previous stage or retire it on threshold breach, performance drift, an unrevalidated model or vendor change, a security incident, or withdrawal of sponsor agreement. A tool may also remain (‘park’) in the supervised-pilot stage indefinitely if acceptance thresholds are not met, and retirement can occur from any stage. Detailed conditions are given in Table 6.

These domains become decision-relevant when combined with the preceding axes. Table 5 translates the framework into a concrete bridge between use case and action, mapping representative use cases—through their evidence maturity, autonomy and trial impact—to a recommended deployment status ranging from research-only to controlled or restricted operational deployment. Figure 4 shows the corresponding stage-gated pipeline (detailed in Table 6). Consistent with lifecycle-oriented guidance for trustworthy AI [18], the framework spans three phases: a pre-deployment phase (intended use, maturity, risk classification, AI-ready data, local validation and vendor qualification); a deployment phase (human oversight, SOPs, workflow integration and documentation); and a post-deployment phase (drift and performance monitoring, incident logging, periodic review and defined update or retirement criteria).

A framework is only useful at a site if it states, without ambiguity, what has to be true before an AI tool may be used on a real trial. We therefore specify minimum conditions for each transition, from research use to a supervised pilot and from a pilot to controlled operational use (Table 6). Three points in that table are deliberately strict. First, a pilot runs in parallel with the existing process and does not replace it, so that the AI can be wrong without harming the trial. Second, acceptance thresholds are agreed before testing begins, because a threshold chosen after seeing the results is not a threshold. Third, the conditions for withdrawing a tool are defined at the same time as the conditions for deploying it; a system that cannot be switched off in a defined way should not be switched on.

A tool that does not meet the conditions for a stage remains at the preceding stage. Autonomous action on eligibility, safety reporting or endpoint adjudication is outside this table and is not recommended.

The site cannot satisfy these conditions alone, and a framework that pretends otherwise will not be implemented. Trial sites operate inside a sponsor–CRO–site relationship in which accountability for trial conduct is defined by GCP and cannot be reallocated by contract [54]. Table 7 therefore sets out who decides, who executes, and who verifies for each readiness domain. Two consequences deserve emphasis. Under GCP, tasks are delegated to people and recorded on a delegation log; an AI system is not a delegatee but a tool used by a delegated person, so accountability does not move to the model or to its vendor. And because a single site typically runs studies for several sponsors with different positions on AI, a site is usually better served by one internal AI policy calibrated to its most restrictive sponsor than by a separate arrangement for each study.

Accountability for trial conduct remains with the sponsor and the investigator under ICH E6(R3) [54]. Nothing in this allocation transfers that accountability to a contract research organization or to a technology vendor.

### 3.11. Regulatory, GCP and Reporting Alignment

The readiness of AI for clinical trial operations must be judged against the regulatory and GCP framework. The FDA draft guidance on AI to support regulatory decision-making introduces a defined context of use and a risk-based credibility assessment [64]. The joint FDA–EMA guiding principles articulate human-centric design, risk-based approaches, data governance, performance assessment and lifecycle management [65]. ICH E6(R3) provides the overarching, technology-agnostic GCP framework for quality by design, data integrity and sponsor/investigator responsibilities [54]. ICH E8(R1), with its emphasis on quality by design and critical-to-quality factors, maps particularly well onto AI readiness understood as quality planning [66]. The EMA reflection paper offers a European lifecycle perspective [67]. The EU Artificial Intelligence Act (in force from 1 August 2024), as amended by Regulation (EU) 2026/1744 (the Digital Omnibus on AI), introduces risk-tiered obligations with a staggered application timetable (general applicability from 2 August 2026; high-risk obligations deferred to 2 December 2027 for Annex III and 2 August 2028 for Annex I systems) [68]. These become relevant wherever operational AI approaches medical-device or high-risk status, and a need for sector-specific guidance in healthcare has been recognized [69]. The treatment of AI as a medical device clarifies the boundary of the present scope [15].

One practical consequence of this alignment is easily overlooked. Operational AI used in a trial is a computerized system, and computerized systems in regulated research already sit inside a mature compliance framework. ICH E6(R3) sets expectations for computerized systems used in trials, including risk-proportionate validation, system release, security, access control, audit trails and data-life-cycle integrity [54]. In the United States, electronic records and signatures relied upon in clinical investigations fall under 21 CFR Part 11 [70], and current FDA guidance addresses electronic systems, records and signatures in clinical investigations specifically [71]. In the European Union, Annex 11 sets corresponding expectations for computerized systems and is itself under revision [72], while risk-based validation of computerized systems in regulated environments is codified in GAMP 5 [73]. Treating AI as an operational layer therefore need not create a new compliance regime; it brings AI inside an existing one. In concrete terms, an AI tool used in trial conduct should have a documented intended use and risk classification, a validation plan and report, defined access control, an audit trail, change control and a documented release for use. These are the artefacts expected of any other regulated computerized system, and they are the artefacts that Box 1 assembles. What is new is not the requirement but its difficulty. A deterministic system can be validated against fixed expected outputs, whereas a generative model may not return the same output twice, and its behaviour may shift as the underlying data or the model itself changes. Validation therefore has to be specified at the level of the AI-enabled workflow, with pre-defined acceptance criteria, a human checkpoint and periodic re-qualification, rather than at the level of a single model output.

The applicability of the EU Artificial Intelligence Act to operational trial AI is narrower, and more specific, than is often assumed [74]. The Regulation does not apply to AI systems developed and put into service for the sole purpose of scientific research and development (Article 2(6)), nor to research, testing or development activity prior to placing on the market (Article 2(8)). Those exclusions cover AI as the object of research. They do not cover AI used as a tool to conduct research: Recital 25 provides that any other AI system that may be used for the conduct of a research activity remains subject to the Regulation. A commercial language model used to prescreen participants is therefore within scope. Whether it is high-risk is a separate question, and on the present text the answer is usually no. The Annex I route requires the system to be a product, or the safety component of a product, covered by sectoral legislation such as the Medical Device Regulation, and operational trial AI is normally not a device on the intended-purpose test [75]. The Annex III list does not clearly capture prescreening of trial participants: its eligibility provision is confined to essential public assistance benefits and services and to systems used by, or on behalf of, public authorities. AI used to monitor or evaluate the performance of site staff, by contrast, would be captured.

The practical consequence is uncomfortable. Most operational trial AI sits inside the AI Act but outside its high-risk regime, so the obligations that actually bite are the AI-literacy duty on deployers, which includes sponsors, contract research organizations and sites, and the transparency duties requiring that a person be told when they are interacting with an AI system, which is directly relevant to participant-facing consent tools. That is a thin set of duties for a technology that can influence who is offered access to a trial. It is one concrete sense in which an operational AI governance gap exists: the instrument most often invoked in discussion of AI in trials imposes, in this setting, less than is commonly assumed, while the instruments that do impose substantive obligations are GCP, data-protection law and the rules for computerized systems.

A further governance gap concerns reporting standards. Established guidelines—SPIRIT-AI and CONSORT-AI for trials of AI interventions [20,21], DECIDE-AI for early clinical evaluation of AI decision-support [22], and TRIPOD + AI for AI prediction models [23]—were designed for studies in which the AI system is the object of evaluation. For AI-based feasibility, site-risk or monitoring models in particular, such reporting standards should be complemented by appraisal tools such as PROBAST + AI, because transparent reporting alone does not establish low risk of bias or local applicability [26]. Most AI tools discussed here are not the intervention; they operate inside the trial as recruitment, monitoring, documentation or safety-triage aids, and no comparable, widely adopted standard yet governs the reporting and qualification of such operational AI workflows. The consistent message is that AI becomes acceptable only when it has a defined context of use, is validated for that context, is auditable, is subject to lifecycle management and remains under meaningful human oversight—precisely the properties the site-level framework is designed to demonstrate at the clinical trial site level. To separate binding obligations from recommended practice, Supplementary Data S1 maps each recommended control to its basis—binding legal or regulatory requirement, guidance or standard, or authors’ recommended practice—together with jurisdictional caveats, and it compares established AI reporting guidelines (SPIRIT-AI, CONSORT-AI, DECIDE-AI, TRIPOD + AI, PROBAST + AI and FUTURE-AI) against their applicability to operational AI inside trial workflows.

### 3.12. Data Protection and Cybersecurity

Data protection is not a side condition of operational AI in trials. For many sites it is the binding constraint, and three questions determine whether an AI-enabled workflow is lawful at all. The first is the allocation of roles. The sponsor is normally a controller; the site or investigator is a controller, or a joint controller, for the source record; a contract research organization ordinarily acts as a processor; and an external AI vendor is a processor only for as long as it processes strictly on documented instructions [76]. The moment a vendor uses trial data to train or improve its own models, it pursues its own purpose and becomes a controller, which requires its own lawful basis for processing health data and disclosure to participants [62]. This cannot be argued away in a data-processing agreement. It has to be contractually excluded and then verified. The European Data Protection Board has addressed data-protection aspects of AI models, including when a model may be considered anonymous and when legitimate interest may be relied upon [77], but we are not aware of guidance addressing third-party AI vendors in clinical trial operations specifically.

The second question is where the data go and where they rest. Sending source data to an external AI service is a transfer, and where the service processes outside the European Economic Area, it engages the transfer rules of the General Data Protection Regulation, which require an adequacy decision or appropriate safeguards together with an assessment of the destination. Data residency, retention and deletion must be specified. A site that cannot state where its prompts are stored, for how long they are kept, and whether they can be deleted, cannot demonstrate compliance. Given the scale of processing and the special category of the data, a data protection impact assessment is in practice mandatory for AI prescreening of trial participants. The third question is automated decision-making. Where an investigator genuinely reviews each candidate, the prohibition on solely automated decisions is normally not engaged. Where a model silently excludes patients who are never surfaced to any human, there is no human decision at all for those individuals; and the Court of Justice of the European Union has held that an automated score may itself constitute the decision where the person acting on it draws strongly upon it [78]. Whether non-enrolment in a trial amounts to a similarly significant effect has not been tested, and we do not assert that it does. But a site that cannot demonstrate meaningful human review is exposed to the question.

Cybersecurity belongs in the same discussion, because an external AI vendor is a supply-chain dependency. Under the NIS2 Directive, healthcare providers and entities carrying out research and development of medicinal products fall within the sectors of high criticality, and the risk-management obligations extend explicitly to supply-chain security and carry incident-reporting timelines [79]. For a trial site, this converts vendor qualification from good practice into a legal expectation. Certification against information-security and AI-management standards, in particular ISO/IEC 27001 [80] and ISO/IEC 42001 [81], is a reasonable procurement signal, although it should not be mistaken for compliance with the AI Act, which has its own conformity route. Looking further ahead, the European Health Data Space will govern the secondary use of health data, including reuse for training and validating models, through data permits and secure processing environments [82]. It does not govern the primary use of data to run a trial, and the two should not be conflated.

## 4. Discussion

This review supports three main conclusions. First, AI in clinical trial operations is genuinely transitioning from isolated task automation toward a workflow-embedded operational layer [1,4,35]. Second, operational maturity is markedly uneven: recruitment and eligibility assessment are comparatively mature [32,33,34,46], whereas patient-facing consent tools, digital twins and synthetic controls remain emerging or high-risk [43,45,48,49]. Third, the barriers to responsible adoption are predominantly organizational and governance-related rather than purely technical [5,7,44].

The review’s contribution is to connect four dimensions rarely considered together—evidence maturity, AI autonomy, trial impact and site readiness—into a single, author-developed and as yet unvalidated deployment-decision aid, situated within the broader movement toward trustworthy, lifecycle-governed healthcare AI [16,17,18,83].

A recurring theme is that implementation readiness cannot be inferred from model performance alone; clinical adoption requires a multi-faceted implementation evaluation rather than accuracy metrics in isolation [84]. The relevant unit of validation is the AI-enabled workflow—the model together with its source data, user role, standard operating procedure, escalation rules, documentation pathway and final human decision—rather than the model in isolation. Validating a model without validating the workflow around it provides false reassurance, because most failure modes in a regulated setting arise at the interfaces between the model and the humans, data and procedures that surround it. Earlier benchmark-style evaluations of large language models on medical examination tasks, including a national medical specialization examination, illustrate this distinction: the ability to answer domain-specific questions is not equivalent to readiness for regulated clinical-workflow deployment [85]. A similar translational-readiness problem has been described in AI-enabled obesity care, where AI was framed as part of a broader operational architecture requiring pathway integration, human oversight and governance rather than predictive performance alone [86].

**Figure 5:**
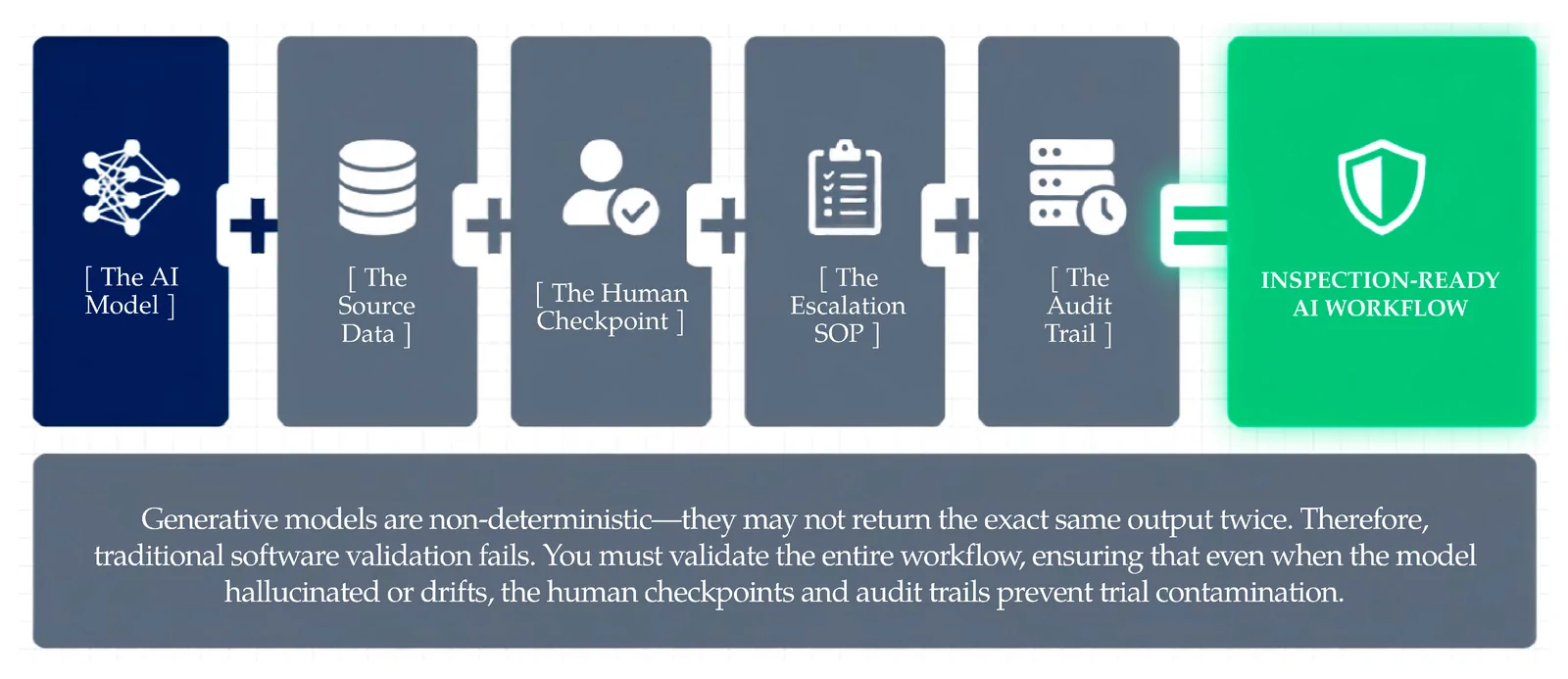
The workflow-validation paradigm. Because a generative model is non-deterministic and may not return the same output twice, traditional software validation of the model alone is insufficient. The unit of validation is the AI-enabled workflow: the model together with defined source data, a human checkpoint, an escalation standard operating procedure, and an audit trail. Validated as a whole, the workflow remains inspection-ready even when the model errs or drifts, because the human checkpoints and audit trail prevent an unreviewed output from contaminating the trial.

This reframing implies a more demanding standard than explainability. For clinical trial operations, the operative requirement is not only explainable AI but inspection-ready AI. At any point, a monitor, auditor, or inspector should be able to reconstruct the following: which model version produced a given output; what data were used; what was generated; who reviewed it; what the final decision was; what entered the trial documentation; and whether the output was modified or rejected. Inspection-readiness maps onto GCP expectations for data integrity and accountability and can be operationalized through the readiness domains, deployment mapping, pathway and dossier proposed here (Table 4 and Table 5, Figure 4, Box 1), and it requires a clear accountability chain across sponsor, site, investigator, vendor, monitor and data-protection roles [83]. The elements that make a workflow inspection-ready are shown in Figure 5.

Framed organizationally, healthcare organizations that host clinical trial sites are not merely physical locations for sponsor-led studies; they are complex adaptive sociotechnical systems in which AI-enabled workflows involve people, processes, technology, governance and organizational culture, not technology alone [25]. They provide the data infrastructure, workforce, privacy controls, quality systems, patient-facing communication channels and governance culture within which AI-enabled trial workflows are implemented. Site-level AI readiness is therefore also an organizational capability, and much of the readiness framework proposed here overlaps with the broader digital-health governance responsibilities of the hosting organization. Consequently, AI may reduce task-level burden while simultaneously increasing system-level governance burden: each efficiency gain introduces new obligations—validation, training, standard operating procedures, audit trails, vendor qualification, data security and drift monitoring—that fall disproportionately on sites and their workforce. Operational readiness is therefore not equivalent to regulatory readiness, and AI readiness in clinical trial operations should be assessed at the level of the workflow, not the model. Consistent with the human–AI teaming evidence, the framework treats AI as decision support that culminates in a documented human decision, not as an autonomous substitute for trial staff [46]. The risk, moreover, is not only that AI tools fail validation, but that they are adopted in pilots and later abandoned because they do not fit site workflows, organizational capacity or sustainability requirements—a pattern well described for health and care technologies more broadly [87].

This review does not claim that the proposed framework is sufficient for regulatory acceptance, nor that AI-enabled workflows should be adopted wherever evidence maturity is higher. Rather, it provides a structured way to identify the minimum site-level conditions that should be satisfied before operational use is considered.

Two contrasting external examples help calibrate expectations. A randomized controlled trial of a generative-AI system for low-dose digital subtraction angiography demonstrates the standard of prospective clinical validation that AI can reach [88]; however, it evaluates AI as a clinical imaging intervention rather than as trial-operational infrastructure, and it therefore illustrates, by contrast, the prospective evidence that operational trial AI still lacks rather than adding to the operational corpus. In an adjacent direction, work on generalized label-free artefact cleaning for real-time physiological time series, implemented within clinical-research monitoring software, is relevant to continuous data-quality and decentralized-monitoring workflows [89], but it is not itself a clinical-trial-operations study and is cited here only as adjacent evidence, not as operational maturity evidence.

### 4.1. Market Enthusiasm Versus Operational Readiness

Commercial and operational data help explain the pace of AI adoption without establishing readiness [9,11,90], and they raise the risk not only of slow adoption but of premature adoption. Consulting and market analyses converge on rapid projected growth of AI in clinical development [12,91,92,93,94], news coverage cautions that automation still requires human oversight [95], and registry snapshots and forecasts of decentralized-trial and AI activity point in the same direction [96,97]; these contextual sources are summarized in the Supplementary Materials and are not treated as evidence of AI effectiveness. A structured comparison of market momentum against evidence maturity by domain is provided in Supplementary Table S3. Three layers follow from it. Market evidence, together with registry-level counts of AI-related trials (Supplementary Figure S1), explains why adoption is accelerating. Peer-reviewed evidence shows where AI is mature or immature. Regulatory, GCP and reporting principles define what must be in place before adoption becomes responsible. The relevant question is therefore whether AI-enabled workflows can be validated, audited and operated safely at the site level.

The market for AI-enabled clinical-trial solutions is expanding rapidly, but market growth should not be confused with operational maturity or regulatory readiness.

### 4.2. Implementation Outcomes for AI-Enabled Trial Workflows

Because clinical adoption depends on more than model performance [84], evaluation should extend to implementation outcomes. Such outcomes—distinct from model-performance outcomes—have been defined to include acceptability, adoption, appropriateness, feasibility, fidelity, implementation cost, penetration, and sustainability [24]. Future evaluations should report implementation outcomes alongside technical metrics: time saved; the additional verification workload created for site staff; the number and proportion of AI outputs overridden or rejected; false-positive and false-negative eligibility flags; time to query resolution; the number of safety alerts escalated; staff acceptability and trust; training burden; equity and unintended consequences; and any inspection findings related to AI use. Reporting these outcomes would move the field from demonstrating that a model can perform a task to demonstrating that an AI-enabled workflow can be operated safely, sustainably and accountably at a site. A consolidated evidence-gap and research agenda is set out in Table 8.

### 4.3. Practical Implications for Healthcare Organizations and Trial Sites

For healthcare organizations and the trial sites they host, several practical implications follow. First, no AI-enabled workflow should be introduced without an explicitly defined context of use and risk classification. Second, AI should not be used for eligibility or safety decisions without a documented human sign-off. Third, models should be validated locally against the site’s own population and workflow before operational use. Fourth, every AI-influenced decision should leave an audit trail sufficient to reconstruct it during monitoring or inspection. Fifth, performance drift and the additional verification workload placed on staff should be monitored after deployment. These implications map directly onto the readiness domains (Box 1) and the deployment pathway (Figure 4) and are intended to be actionable at the level of a research-governance function. For a coordinator or investigator, they are often felt as small but concrete changes to daily work—for example, a required sign-off field in the screening system before a prescreened patient is entered into the screening log, or a note in the trial master file recording which model version produced a flagged eligibility match.

Two further constraints determine whether any of this is actually done. The first is that a site is rarely a free agent. A single site may run many trials for several sponsors, each with its own position on AI, while the sponsor retains oversight of trial conduct and of delegated activities [54]. The use of an AI tool in a given trial should therefore be agreed with the sponsor before it is used. It should also be recorded in the investigator site file and trial master file and reflected in the delegation and training records of the staff who operate it. The second constraint is cost. Local validation, vendor qualification, training, additional verification steps, and drift monitoring consume staff time that is not currently a line item in most clinical trial agreements or site budgets. Where these activities are neither funded nor assigned to a named role, they are unlikely to be performed. Efficiency gained at the level of the model may then be offset, or even reversed, at the level of the site. This concern is consistent with reports of rising site burden [3], although we are not aware of empirical data quantifying the net effect of AI adoption on site workload.

These constraints have a health-economic dimension that the current evidence base does not yet allow us to quantify. Model performance is not the same as total cost of ownership: an AI-enabled workflow carries costs for local validation, vendor qualification, training, additional verification steps, documentation and lifecycle monitoring, set against potential savings in screening time, query resolution and database-lock timelines. Because the published operational evidence does not report these quantities, we did not attempt a cost-effectiveness analysis. We instead recommend that future implementation studies report implementation and compliance costs, staff verification workload, training burden, time saved and screen-failure impact alongside performance, so that the economic case for site-level adoption can be assessed rather than assumed.

### 4.4. Site Typology and Implementation Capacity

Site-level AI readiness is not uniform. The same AI-enabled workflow may be feasible in a large academic medical center with an informatics team and effectively inaccessible to a small community site that depends entirely on sponsor-provided systems. The readiness framework should therefore be applied with attention to site typology and implementation capacity (Table 9). Large academic or integrated health-system sites may be able to conduct local validation, maintain data pipelines and review model performance internally. Mid-size sites may need sponsor- or contract-research-organization-provided validation packages, clear documentation exports and simplified standard-operating-procedure templates. Small or community sites may require highly restricted use cases, centrally provided validation evidence, minimal additional system burden and explicit sponsor support for documentation, training and incident handling. Site typology does not change the need for GCP-aligned oversight, but it changes what can realistically be performed locally and what must be supplied by the sponsor, CRO or vendor.

### 4.5. Sponsor-Mandated AI Workflows and Site–Sponsor Negotiation Points

Many trial sites will not choose AI-enabled workflows themselves. More often, sponsors or CROs deploy AI-enabled monitoring, prescreening, documentation, or safety tools and expect the site to participate. This creates an asymmetry: the site remains accountable for GCP-compliant conduct and source documentation, yet it may not control the model, the validation evidence, the update schedule or the audit-log export. Before accepting a sponsor-mandated AI workflow, a site should therefore request a minimum AI documentation package and agree operational boundaries before first use (Table 10). Framing these as explicit negotiation points, rather than post-hoc concerns, allows the site to file the necessary evidence in the investigator site file and trial master file and to retain a defined route to suspend use.

### 4.6. Limitations

As a structured narrative review, this work does not provide an exhaustive, protocol-driven search or a quantitative synthesis and is subject to selection bias and to the rapid evolution of the field. The evidence-maturity score is a pragmatic mapping tool, not a validated instrument. The readiness framework, deployment mapping and pathway should likewise be read as a conceptual implementation aid rather than a validated tool. Future empirical work should test whether the framework improves implementation quality, audit-readiness, staff confidence, participant safety or inspection outcomes at real sites. In particular, the framework was not developed through a Delphi process, stakeholder consultation or empirical testing with site personnel, sponsors, monitors or regulators. The evidence supporting the strongest maturity rating also has a linguistic and geographical boundary. The recruitment and eligibility systems reviewed here were developed and evaluated predominantly on English-language clinical records and in a small number of centers. Source documents at many trial sites are written in the local language and use local abbreviations, templates and coding practices. Whether the reported performance transfers to non-English electronic health records has not been demonstrated, so these findings should not be assumed to generalize across languages and documentation cultures without local validation. Database yields are reported for PubMed/MEDLINE only; the other sources were used for citation chaining and grey-literature retrieval rather than as separately counted search streams, and this limits the reproducibility of the search beyond the primary database. Some supporting evidence derives from preprints or adjacent literatures such as pharmacovigilance and is used with caution, and market and registry figures are used only as contextual signals. The regulatory and data-protection analysis reflects the position at the time of writing, including Regulation (EU) 2026/1744, which amended the AI Act and deferred the high-risk application dates; because implementing guidance continues to develop, readers should verify the current position before relying on it. Further research should also develop standardized reporting for operational AI workflows and invest in AI literacy for the research workforce.

## 5. Conclusions

Artificial intelligence is moving from isolated task automation toward an operational layer that can influence how clinical trials are designed, recruited, monitored, documented, and overseen. Its greatest current value lies in recruitment and eligibility assessment, where empirical evidence is strongest, while other applications remain emerging or context-dependent. Extending prior lifecycle-level frameworks, the distinctive contribution of this review is an author-developed, unvalidated site-level readiness framework and preliminary deployment-decision aid linking evidence maturity, technical validity, AI autonomy, trial impact and site implementation capacity to operational use. The framework should be understood as a synthesis-derived implementation checklist for further testing, not as a validated regulatory standard. Across all use cases, technical performance is necessary but insufficient for readiness, which also depends on operational validation, governance, auditability, and human oversight. AI in clinical trial operations should therefore be treated as a managed, inspection-ready and GCP-aligned operational layer, not as an autonomous substitute for investigators and trial staff. In practice, this means validating the whole workflow for a defined context of use, keeping it auditable, monitoring it across its lifecycle and embedding it in human accountability. Much of this does not require a new compliance regime so much as the disciplined application of an existing one. In clinical trial operations, AI readiness should be assessed at the level of the workflow, not the model, and closing that gap at the level of the trial site and its host healthcare organization is the central practical challenge for the responsible adoption of AI in clinical research.

## Figures and Tables

**Table 1:** Evidence-maturity scoring rubric (0–8). Class bands: 0–2 emerging; 3–4 limited; 5–6 moderate; 7–8 high. A score of 7–8 requires prospective or real-world evaluation, multi-site or external data, testing inside an operational workflow, and explicit governance reporting; no use case in this corpus met that standard.

Criterion	0 Points	1 Point	2 Points
Evidence type	Conceptual/preprint	Retrospective/technical validation	Prospective/user/real-world evaluation
Data realism	Synthetic/demonstration	Single-centre real-world	Multi-site/external data
Workflow integration	Offline model only	Simulated workflow	Operational workflow testing
Governance reporting	Absent	Partial	Explicit oversight/auditability/validation

**Table 2:** AI applications across the clinical trial lifecycle.

Stage	AI Use Case	Potential Value	Main Risk	Required Governance
Protocol design	Eligibility simplification, protocol assessment	Better feasibility, reduced complexity	Misinterpretation, hallucination	Expert review, version control
Feasibility/site selection	Patient-pool, site and investigator ranking	Better site choice, realistic recruitment	Historical/data bias	Transparent criteria, local validation
Recruitment/eligibility	Patient-trial matching, criterion-level screening	Faster screening, better matching	False positives/false negatives	Human verification, audit trail
Conduct/monitoring	RBQM, deviation detection, site risk scoring	Proactive oversight	Alert fatigue, missed issues	SOP thresholds, escalation pathways
Data management	Data cleaning, query generation	Reduced site burden, faster database lock	Erroneous queries	Human review, traceability
Safety	Adverse-event/ADE signal support	Earlier signal detection	Over- or under-reporting	Safety physician review
Consent/education	Consent-form generation, chatbot support	Readability, accessibility	Hallucination, undue influence	Approved content boundaries, escalation

**Table 3:** Evidence-maturity map of AI use cases (scored per Table 1; subscores in Supplementary Table S2).

Use Case	Score (0–8)	Class	Evidence Basis	Key Sources
Recruitment/eligibility	6	Moderate	Multiple empirical systems; randomized human–AI teaming evaluation on retrospective data; not yet tested inside a live trial workflow	[28,32,33,34,46]
Site selection/feasibility	5	Moderate	ML ranking and externally validated site-risk models; no prospective operational deployment	[29,30,31]
Safety surveillance/ADE	4	Limited	Largely offline benchmarks and reviews from pharmacovigilance rather than trial conduct	[39,40,41,47]
RBQM/monitoring/deviations	3	Limited	Scoping review plus targeted methods papers; no prospective evaluation	[35,36]
Data management/cleaning	3	Limited	Efficiency evidence including a preprint; governance reporting absent	[37,38]
Patient-facing consent AI	3	Limited	Early LLM/RAG evaluations of high-stakes communication	[42,43,45]
Digital twins/synthetic controls	2	Emerging	Conceptual and review evidence; limited routine use	[48,49,50]

**Table 4:** AI autonomy, trial impact and governance intensity.

Axis and Level	Description/Example	Governance Requirement
Autonomy—L1 (administrative support)	Protocol summary, draft communication	Human review, version control
Autonomy—L2 (decision support)	List of potentially eligible patients	Documented final human decision, validation, override rule
Autonomy—L3 (semi-automated)	AI prescreening with escalation rules	SOP, thresholds, audit trail, escalation pathway
Autonomy—L4 (autonomous action)	AI includes/excludes a participant without review	Generally not recommended for critical tasks without exceptional validation and regulatory justification
Trial impact—Low	Internal administrative summaries, scheduling, non-regulatory drafts	Light-touch human review
Trial impact—Moderate	Prescreening lists, query prioritization, site risk alerts	Documented human decision, validation
Trial impact—High	Eligibility support, consent communication, safety triage, regulatory documentation	Validation, audit trail, explicit oversight
Trial impact—Critical	Autonomous inclusion/exclusion, SAE reporting decisions, endpoint adjudication	Generally not delegated to AI without exceptional justification

**Table 5:** From AI use case to site-level deployment recommendation (a synthesis-derived aid, not a validated instrument).

Use Case	Evidence Maturity	AI Autonomy	Trial Impact	Deployment Recommendation
Patient–trial matching/eligibility	Moderate	Decision support	Moderate–high	Controlled pilot with mandatory human verification; not autonomous inclusion/exclusion
Site selection/feasibility	Moderate	Decision support	Moderate	Decision support only; transparent criteria and local validation
RBQM/risk alerts	Limited	Semi-automated alerting	Moderate–high	Risk alerting only; no autonomous oversight
AI-assisted data cleaning	Limited	Semi-automated	Moderate	Human-reviewed and audit-trailed; validated pipeline
AI-generated consent/education	Limited	Administrative support	High	Bounded content, IRB/legal review, disclosure and escalation
Safety/ADE triage	Limited	Decision support	High	Triage/prioritization layer under safety-physician review
Digital twins/synthetic controls	Emerging	Decision support	High	Research use only; not routine site workflow

**Table 6:** Minimum conditions for moving an AI-enabled workflow from research use to pilot and to controlled operational use.

Stage	Evidence and Validation	Error Tolerance	Human Oversight	Exit/Rollback Criteria
Research use only	Published or internal evidence of any maturity; no local validation required	Not applicable: outputs must not influence any trial decision or enter trial documentation	Outputs are not used in trial conduct	Not applicable
Supervised pilot (parallel run)	Local validation on the site’s own retrospective data against a documented reference standard; acceptance thresholds, covering both technical-performance and operational-safety criteria, agreed with the sponsor before testing begins	Pre-specified and impact-dependent. For eligibility, the tolerated number of ineligible participants entering the screening log is zero; model sensitivity and specificity are reported and compared with the site’s current process. For moderate-impact tasks, thresholds are set locally and justified	For high-impact outputs (eligibility, safety, consent), 100% human review of every AI output; for moderate-impact outputs (e.g., query prioritization, data cleaning, site-risk alerts), justified documented sampling may be used with full review of all flagged and discordant cases. In all cases the AI runs alongside the existing process and does not replace it	Pilot stops if acceptance thresholds are not met, if any unreviewed output reaches trial documentation, or if an eligibility- or safety-relevant error occurs
Controlled operational use	Acceptance thresholds met in the pilot and confirmed on a prospective sample at the site; released for use under change control	Continuous monitoring against the same pre-specified thresholds; a breach reverts the tool to pilot conditions	Risk-proportionate: full review for high-impact outputs; documented sampling for moderate-impact outputs, with full review of all flagged and all discordant cases	Defined triggers for suspension and retirement: threshold breach, performance drift, model or vendor change without revalidation, security incident, or withdrawal of sponsor agreement

**Table 7:** Allocation of responsibility for AI-enabled trial workflows across site, sponsor, CRO and technology vendor.

Readiness Domain	Site/Investigator	Sponsor	CRO	Technology Vendor
Intended use and risk classification	Proposes the use case; the investigator confirms clinical relevance and acceptability	Agrees to the use for the specific trial; confirms fit with the protocol	Advises; reflects the agreed use in the monitoring plan	States the intended purpose, the operating limits and the known failure modes
Data protection and legal basis	Controller or joint controller for the source record; performs the impact assessment	Controller; determines the purposes of processing	Processor for the sponsor, unless acting for its own purposes	Processor only; no training or model improvement on trial data without a separate lawful basis
Local validation	Executes validation on its own data and population	Reviews and accepts the validation report	May perform validation on the sponsor’s behalf	Supplies performance data, model and version information, and known limitations
Human oversight	The investigator remains accountable; oversight is not delegable to a system	Defines the minimum oversight required in the protocol and monitoring plan	Verifies at monitoring visits that the required review actually occurred	Provides override, escalation and audit functions
Documentation and audit trail	Files the dossier in the investigator site file; retains the decision log	Files in the trial master file; retains accountability for records	Ensures trial master file completeness	Guarantees an audit trail and the export of records
Training and delegation	Trains staff; AI-related tasks appear in the delegation and training records	Confirms at the site initiation visit	Checks training records during monitoring	Provides training materials and version-specific documentation
Incidents and CAPA	Reports AI errors as quality issues within the existing quality management system	Performs root-cause analysis; owns corrective and preventive actions	Escalates and tracks to closure	Reports defects, model changes and security incidents
Lifecycle and retirement	Monitors drift; suspends the tool when a trigger is met	Approves continued use; approves retirement	Reports performance and incidents through monitoring	Notifies model updates, deprecation and end of support

**Table 8:** Evidence gaps and research agenda for AI-enabled clinical trial operations.

Domain	Current Evidence Gap	Recommended Study Design	Minimum Outcomes to Report
Eligibility matching	Limited prospective site-level validation	Prospective multi-site implementation study	Accuracy, screen-failure rate, staff workload, override rate
Consent AI	Limited participant-level evidence	Controlled comprehension and usability study	Comprehension, trust, escalation, undue influence
RBQM/risk alerts	Limited impact on monitoring quality	Prospective operational evaluation	False-alert rate, missed issues, deviation detection
Data cleaning	Preprint- and efficiency-heavy evidence	Controlled workflow evaluation	Query accuracy, time saved, human-correction rate
Safety triage	Uncertain transfer from pharmacovigilance	Trial-specific safety-workflow study	Escalation appropriateness, false-negative rate
Digital twins/synthetic data	Limited routine operational use	Regulatory-grade validation study	Validity, bias, acceptability, regulatory acceptance

**Table 9:** Site typology and its implications for AI-readiness assessment.

Site Type	Typical Capacity	Readiness Implications	Main Caution
Large academic/integrated health-system site	Internal informatics, quality and research-governance capacity; access to EHR data	Can execute local validation, maintain an AI-use dossier and monitor drift locally	Avoid over-customization; ensure sponsor acceptance of local validation evidence
Mid-size hospital or commercial site	Operational research team but limited data-science capacity	Needs sponsor/CRO validation package, model and version documentation, clear SOP and audit-trail export	Do not accept a black-box sponsor tool without a validation and documentation package
Small/community site	Limited IT and quality resources; high staff burden	Restrict to low- or moderate-impact decision support; require sponsor-provided validation, training and support	Avoid high-impact AI workflows unless the sponsor assumes documentation, training and monitoring support

**Table 10:** Site–sponsor negotiation points before accepting a sponsor-mandated AI-enabled workflow.

Domain	Question for the Sponsor, CRO or Vendor	Expected Documentation to Receive and File
Intended use	What task will the AI support, and what decisions may it influence?	Protocol note, work instruction, monitoring plan or site-file memo
Data processed	Which source, trial or participant-facing data will be processed, and where?	Data-flow map and data-protection assessment
Technical and operational validation	What performance, error, fairness and local-applicability evidence exists?	Validation summary, stated limitations, acceptance thresholds
Model/version control	Which model version is used and how are updates notified?	Version record, change-control process, revalidation trigger
Human oversight	Who reviews, accepts, rejects or overrides AI outputs?	Decision log, delegation and training records, SOP
Documentation location	What must be filed in the ISF and TMF?	AI-use dossier, audit-log export, vendor documentation
Incident/CAPA	Who owns root-cause analysis after an AI-related error?	Incident report, CAPA responsibilities, escalation route
Suspension rights	Can the site suspend use after a threshold breach or safety concern?	Rollback and retirement criteria agreed with the sponsor

## Data Availability

No original patient-level or experimental data were generated. The evidence-extraction table, source classification, component maturity scores, scoring rationale, methodological-concern coding, technical-validity extraction, and sensitivity analyses generated for this review are provided as Supplementary Data S1. Contextual market and registry sources are provided in the Supplementary Materials.

## Supplementary Materials

The following supporting information can be downloaded at: https://www.mdpi.com/article/10.3390/healthcare14162519/s1. Table S1. Contextual market and operational sources; Table S2. Evidence-maturity subscores (0–8); Table S3. Market momentum versus evidence maturity; Table S4. Search strategy, database yields and eligibility criteria; Table S5. Characteristics of the principal evidentiary sources; Table S6. Sensitivity of the maturity ranking to alternative weightings; Figure S1. AI-related trial activity in registries; Figure S2. Literature identification, selection and classification; Figure S3. AI Act applicability decision aid for operational trial AI; Supplementary Data S1. Nine-sheet evidence-mapping workbook (source classification, workflow scoring, sensitivity, technical validity, site typology, sponsor negotiation, regulatory basis, framework comparison, reviewer tracker).

## Abbreviations

AI, artificial intelligence; ML, machine learning; LLM, large language model; RAG, retrieval-augmented generation; EHR, electronic health record; RBQM, risk-based quality management; ADE, adverse drug event; DHT, digital health technology; SOP, standard operating procedure; GCP, Good Clinical Practice; FDA, U.S. Food and Drug Administration; EMA, European Medicines Agency; ICH, International Council for Harmonisation; OMOP CDM, Observational Medical Outcomes Partnership Common Data Model; DCT, decentralized clinical trial.
