# Supplementary material for: Artificial Intelligence Readiness in Clinical Trial Operations: A Narrative Review and Site-Level Governance Framework
Source: Healthcare (Basel). 2026 Aug 12;14(16):2519. doi: 10.3390/healthcare14162519 (PMC13511858; doi:10.3390/healthcare14162519)
Supplement: Supplementary file 1 [file healthcare-14-02519-s001.zip › Supmat08.08.pdf]

Supplementary Materials

Artificial Intelligence Readiness in Clinical Trial Operations: A Narrative Review and Site-Level Governance Framework

Supplementary Material

The Supplementary Material provides contextual market and registry sources (Table S1), the evidence-maturity subscores (Table S2), market momentum versus evidence maturity (Table S3), the search strategy, database yields and eligibility criteria (Table S4), characteristics of the principal evidentiary sources (Table S5), the sensitivity analysis of the maturity ranking (Table S6), and illustrative registry, literature-flow and AI Act figures (Figures S1–S3). The full evidence-mapping dataset — source classification, granular workflow scoring, technical-validity extraction, sensitivity analyses, site-typology material, the sponsor-negotiation checklist, the regulatory-basis mapping and the framework comparison — is provided separately as Supplementary Data S1. Contextual sources describe the operational and commercial pressures driving AI adoption; they are not evidence of AI effectiveness, which rests on the peer-reviewed literature cited in the main text. All supplementary references are included in the main reference list.

Supplementary Table S1. Contextual market and operational sources

Source	Year	Area	Key figure (verification status)	Use	Caution
IQVIA, Global R&D Trends [41]	2026	R&D productivity	"Operationally addressable cycle-time" theme (confirmed)	Introduction context	Qualitative framing
Tufts CSDD / Applied Clinical Trials [42]	2024	Cost of delay	~USD 40,000/day mean; Phase III USD 55,716/day (confirmed)	Intro & Discussion	Direct cost only
SCRS Global Site Landscape Survey [43]	2025	Site pressure	Rising costs/complexity (theme confirmed)	Site-readiness rationale	Survey; self-report
CISCRP Perceptions & Insights [44]	2025	Patient perceptions	12,887 respondents (confirmed)	Patient-facing AI	Perception survey
Grand View Research [45]	2025	Market size	USD 89.0B (2025) → 158.4B (2033), CAGR 7.7% (confirmed)	Sector scale (1 sentence)	Commercial forecast
MarketsandMarkets [46]	2024	AI market	USD 1.35B (2024) → 2.75B (2030), CAGR 12.5% (confirmed)	Forecast divergence	Commercial forecast
Fortune Business Insights, AI in CT [47]	2025	AI market	Figures vary by report version — not cited numerically	Forecast divergence	Discrepancy; verify

Fortune Business Insights, DCT [48]	2025	DCT market	USD 9.56B (2025) → 29.37B (2034), CAGR 13.2% (confirmed)	Decentralized data context	Commercial forecast
McKinsey, Faster smarter trials [49]	2025	gen AI value	>USD 50B/yr potential (confirmed)	Discussion (collective)	Consulting estimate
BCG, Agentic AI in Biopharma [50]	2025	GenAI market	~USD 1B → 22B by 2027 (confirmed)	Discussion (collective)	Consulting estimate
Deloitte, Tech Trends 2025 [51]	2025	Tech trends	AI reshaping biopharma (confirmed)	Discussion (collective)	Consulting outlook
Accenture, Reinventing R&D [52]	2025	R&D acceleration	~4 yrs faster, 35–45% cost cut (vendor-adjacent)	Discussion (collective)	Vendor-adjacent claim
Reuters, CRO/AI selloff [53]	2026	Market/CRO	Human oversight still needed (confirmed via syndication)	Discussion (context)	News item
Kwon [54]	2025	Registry (WHO ICTRP)	596 AI trials, 62 countries; China 35.6%, USA 8.5%	Registry evidence (main text)	Peer-reviewed
Maru et al. [55]	2025	Registry (CT.gov)	17.7% AI/ML studies disclosed within 3 yrs	Registry evidence (main text)	Peer-reviewed
ClinicalTrials.gov snapshot [56]	2026	Registry keyword count	AI 3,912; LLM 366 (own query, 9 Jul 2026)	Supplementary Fig S1 only	Illustrative, non-additive

Supplementary Table S2. Evidence-maturity subscores (0–8)

Use case	Ev. type	Data	Workflow	Gov.	Total	Class	Rationale	Main uncertainty
Recruitment / eligibility	2	2	1	1	6	Moderate	Randomized human–AI teaming evaluation and user study; EHR validation across datasets. Workflow scored 1, not 2: evaluations were user studies or simulated screening, not use inside a live trial	No evaluation inside a live trial workflow
Site selection / feasibility	1	2	1	1	5	Moderate	Externally validated site-risk models on real-world data;	No prospective operational deployment

							evidence type scored 1 because validation is retrospective	
Safety surveillance / ADE	1	2	0	1	4	Limited	Real-world pharmacovigilance data, but predominantly offline benchmarks, reviews and scoping reviews	Transfer from pharmacovigilance to interventional trial conduct
RBQM / monitoring / deviations	1	1	0	1	3	Limited	Scoping review plus targeted methods papers; evidence type scored 1 because no prospective evaluation was identified	Effect on monitoring quality unknown
Data management / cleaning	1	1	1	0	3	Limited	Efficiency evidence including a preprint; governance reporting absent	Peer-reviewed confirmation pending
Patient-facing consent AI	1	1	1	0	3	Limited	Early LLM / RAG evaluations of high-stakes communication	Participant trust, empathy, liability
Digital twins / synthetic controls	1	1	0	0	2	Emerging	Conceptual and review evidence with regulatory uncertainty	Limited routine site-level use

Scoring per Table 1 (main text). Ratings reflect the dominant body of evidence for each use case rather than any single study; they are a transparent mapping aid, not a validated instrument. Top-tier evidence-type subscores were assigned only where prospective, user or real-world evaluations were available (e.g., recruitment and site-selection studies); safety-surveillance evidence draws mainly on real-world pharmacovigilance data (data realism 2) but from retrospective or review sources (evidence type 1).

Supplementary Table S3. Market momentum versus evidence maturity

**Domain**	**Market signal**	**Evidence maturity**	**Governance consequence**
AI in clinical trials (overall)	Rapid but highly divergent forecasts [46,47]	Uneven; strongest for recruitment/eligibility	Growth is not readiness; adopt use-case by use-case
Recruitment / matching	High commercial interest	Moderate	Pilot with mandatory human review
Monitoring / RBQM	Growing vendor activity	Limited	Risk alerting only, not autonomous oversight
Patient-facing AI	Heavily marketed	Limited	Bounded content, escalation, disclosure
Decentralized trials / DHT	Strong DCT market growth	Enabling context; data-quality concerns	New data streams require governance

Digital twins / synthetic data	Framed as the future	Emerging	Research use, not routine site operations

Supplementary Table S4. Search strategy, database yields and eligibility criteria

Query / source	Search string or approach	Yield
PubMed/MEDLINE — broad query (executed 13 July 2026)	("artificial intelligence"[tiab] OR "machine learning"[tiab] OR "large language model*"[tiab] OR "generative AI"[tiab]) AND ("clinical trial*"[tiab] OR "eligibility criteria"[tiab] OR "patient-trial matching"[tiab] OR "trial matching"[tiab] OR "risk-based monitoring"[tiab] OR "protocol deviation*"[tiab] OR "clinical data management"[tiab] OR "trial recruitment"[tiab]) AND 2020/01/01:2026/12/31[dp] AND english[la]	6,877 records
PubMed/MEDLINE — operationally specific query (executed 13 July 2026)	("artificial intelligence"[tiab] OR "machine learning"[tiab] OR "large language model*"[tiab] OR "generative AI"[tiab]) AND ("eligibility criteria"[tiab] OR "trial recruitment"[tiab] OR "trial matching"[tiab] OR "risk-based monitoring"[tiab] OR "protocol deviation*"[tiab] OR "clinical data management"[tiab]) AND 2020/01/01:2026/12/31[dp] AND english[la]	815 records (operationally specific candidate search set; not a fully screened eligible pool)
Scopus; Web of Science Core Collection; Google Scholar	Used for citation chaining from included records and for grey literature; not run as separately counted search streams	Not counted
FDA / EMA / ICH / EUR-Lex	Targeted retrieval of guidance and legal instruments (context of use; good AI practice; E6(R3); E8(R1); AI reflection paper; AI Act; GDPR; NIS2)	Documents retrieved by hand

Inclusion criteria: peer-reviewed empirical studies, reviews, regulatory, legal or reporting-guidance documents and implementation-science frameworks addressing AI applied to the conduct of clinical trials. Exclusion criteria: AI evaluated as a clinical intervention, diagnostic or prognostic model; AI for drug discovery, molecular design or preclinical research; recruitment or eligibility papers with no AI component; AI outside clinical trial operations; trial reports using machine learning only as an outcome-analysis method; editorials, letters and conference abstracts without substantive content. Selection within the eligible pool was purposive, not exhaustive; a PRISMA-style count-based screening flow was not applied; the 815 records are reported as a candidate operational search set, not a fully screened eligible pool.

Supplementary Table S5. Characteristics of the principal evidentiary sources

The table characterizes the principal empirical, review, regulatory and implementation-science sources used for evidence-maturity and governance conclusions.

Contextual market and registry sources are characterized separately in Supplementary Table S1.

Ref.	Source type	Lifecycle stage	Use case	Validation setting	Governance relevance
[9] Jin	Empirical system / user study	Recruitment / eligibility	Patient–trial matching (TrialGPT)	Criterion-level matching; user study	Human verification; audit trail
[10] Abdallah	Empirical system	Recruitment	Patient-to-trial matching (TrialMatchAI)	Structured + unstructured data	Explainability; local deployment
[11] Gupta	Empirical system	Recruitment / EHR	Semantic matching (PRISM)	Real-world EHR validation	Privacy; local validation
[12] Parikh	Randomized evaluation	Eligibility prescreening	Human–AI teaming	Retrospective EHR RCT	Human review; accountability
[14] Datta	Empirical NLP	Feasibility / eligibility	Criteria extraction (AutoCriteria)	Extraction evaluation	Protocol structuring; audit
[15] Lee	Empirical evaluation	Eligibility → query	Criteria-to-query conversion	Technical validation	Hallucination control; auditability
[17] Hulstaert	Empirical ML	Site selection	Site ranking	Real-world data modeling	Transparent criteria; validation
[18] Yang	ML development / ext. validation	Site risk / RBQM	Site-risk prediction	External validation	Transparent criteria; oversight
[7] Teodoro	Scoping review (142)	Risk assessment / RBQM	AI risk prediction	Evidence synthesis	Validation; oversight
[22] Spyroglou	Empirical methods	Protocol deviations	Outlier + generative-AI analysis	Methods evaluation	Proactive site oversight
[23] Shi	Mixed-methods study	Consent	Consent generation	LLM evaluation	Bounded content; IRB review
[24] Giorgi	Empirical system	Consent (conversational)	Consent assistant (RESPECT)	Safety / utility evaluation	Source grounding; escalation
[25] Moscatel	Empirical evaluation	Consent Q&A	Consent process	LLM evaluation	Human oversight; empathy
[28] Mao	Systematic review (262)	Decentralized / DHT	DHT data streams	Evidence synthesis	Data quality; oversight
[35] Schreier	Scoping review (49)	Safety / ADE	ADE analysis	Evidence synthesis	Human safety review
[57] Mateen	Framework	Whole lifecycle	AI adoption framework	Conceptual	Lifecycle governance
[62] You	Framework	Implementation	Real-world AI deployment	Conceptual	Phased deployment
[63] Lekadir	Consensus guideline	Healthcare-AI lifecycle	Trustworthy AI (FUTURE-AI)	Consensus process	Design-to-monitoring governance

[64] van de Sande	Perspective	Implementation evaluation	Multi-faceted evaluation	Conceptual	Implementation outcomes
[65] Domagalski	Methodology	Data readiness	AI-ready data	Data-centric challenge	Data governance
[38] FDA / EMA	Regulatory principles	Drug-development AI	Good AI practice	Guidance	Context of use; lifecycle
[39] ICH E6(R3)	Regulatory guideline	Trial conduct	Good Clinical Practice	Guidance	Data integrity; responsibilities
[79] Moons	Appraisal tool	Prediction models	Risk-of-bias appraisal (PROBAST+AI)	Tool	Bias; applicability
[80] Greenhalgh	Framework	Technology adoption	Non-adoption / scale-up (NASSS)	Conceptual	Sustainability

Supplementary Table S6. Sensitivity of the maturity ranking to alternative weightings Class bands (0–8): 0–2 emerging, 3–4 limited, 5–6 moderate, 7–8 high (as in Table 1). Base scores use equal weighting of the four criteria (evidence type, data realism, workflow integration, governance reporting; each 0–2, sum 0–8). Each alternative column doubles the weight of one criterion and renormalizes to the 0–8 scale (the double-weighted 0–10 sum × 0.8) so that all scores share the same band definitions. Under every weighting the ranking is stable and no use case reaches the high band (≥7); the maximum is recruitment/eligibility at 6.4 under evidence-type weighting. Per-source and per-workflow scores underlying this table are in Supplementary Data S1 (sheets 'Workflow scoring' and 'Sensitivity').

Use case	Equal weights (0–8)	Evidence-type weighted (normalized, 0–8)	Workflow-integration weighted (normalized, 0–8)	Rank stability
Recruitment / eligibility	6	6.4	5.6	Highest under all weightings; remains moderate
Site selection / feasibility	5	4.8	4.8	Rank 2 under all weightings; stable
Safety surveillance / ADE	4	4.0	3.2	Rank 3; stable
RBQM / monitoring / deviations	3	3.2	2.4	Stable; limited band under all weightings
Data management / cleaning	3	3.2	2.4	Stable; limited band under all weightings
Patient-facing consent AI	3	3.2	3.2	Rank stable; rises slightly under workflow weighting but stays in the limited band
Digital twins / synthetic controls	2	2.4	1.6	Lowest under all weightings; emerging

The two most mature use cases retain their rank under both alternative weightings. Under workflow weighting, data cleaning and patient-facing consent AI rise into the same band as safety surveillance. No use case reaches the high band (7–8 on the equal-weight scale) under any weighting. The substantive conclusions are therefore not an artefact of equal weighting.

Supplementary Figure S1. AI-related trial activity in registries

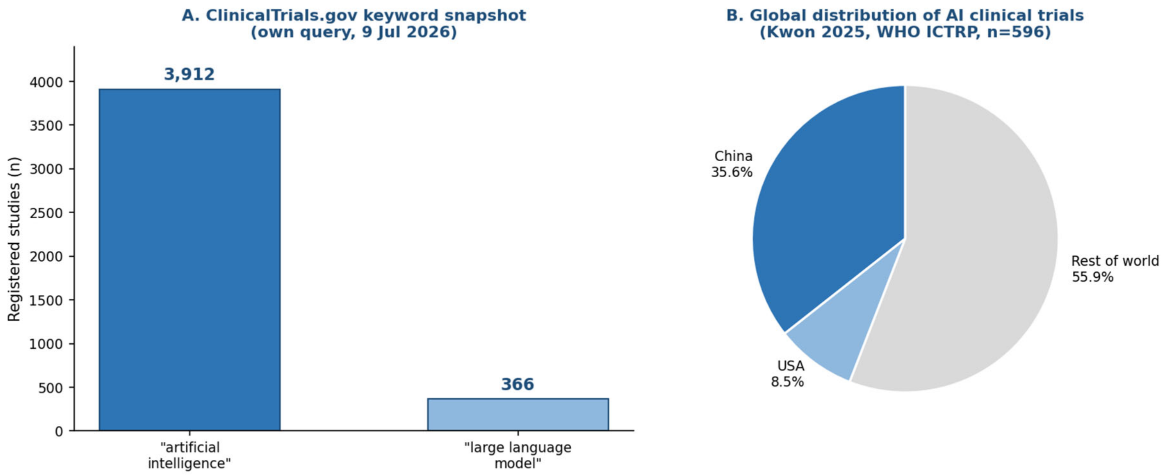

Supplementary Figure S1. (A) Descriptive keyword snapshot of ClinicalTrials.gov (own query, 9 July 2026): studies matching "artificial intelligence" (n = 3,912) versus "large language model" (n = 366); counts are illustrative and non-additive. (B) Global distribution of AI clinical trials from a WHO ICTRP registry analysis (n = 596) [54]. A companion BibTeX file (AI_CT_references.bib) contains all references for automatic citation management.

Supplementary Figure S2. Literature identification, selection and classification

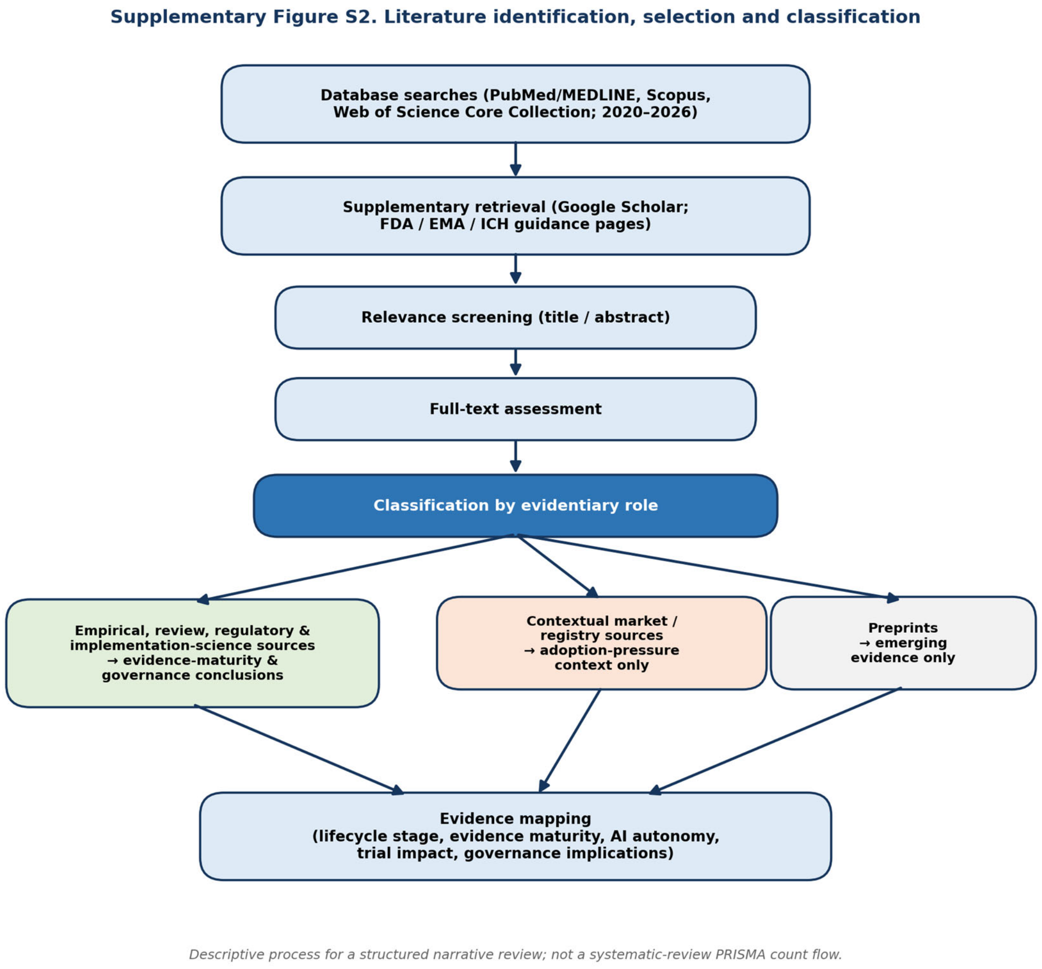

Supplementary Figure S2. Structured narrative-review process: database and supplementary searches, relevance screening, full-text assessment, classification by evidentiary role, and evidence mapping. Descriptive process, not a systematic-review PRISMA count flow.

Supplementary Figure S3. AI Act applicability decision aid for operational trial AI

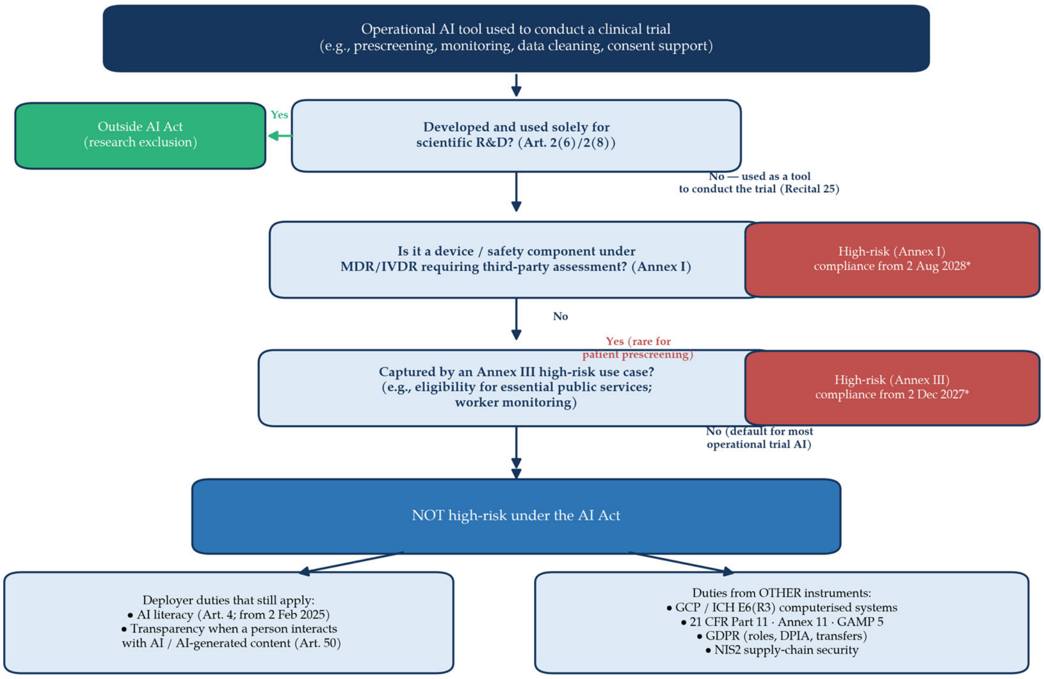

** Application dates reflect Regulation (EU) 2024/1689 as amended by Regulation (EU) 2026/1744 (Digital Omnibus on AI; OJ L, 2026/1744, 24 July 2026). General applicability of the AI Act: 2 August 2026. Classification depends on intended purpose, use case and jurisdiction; this aid is illustrative and does not constitute legal advice.*

Supplementary Figure S3. Simplified decision aid mapping common operational trial-AI use cases to EU Artificial Intelligence Act categories. Most operational trial AI falls inside the Regulation but outside its high-risk regime, leaving AI-literacy and transparency duties under the AI Act and substantive obligations under GCP, computerised-systems rules, data-protection law and cybersecurity law. The aid is illustrative and not legal advice; classification depends on intended purpose, use case and jurisdiction, and application dates reflect Regulation (EU) 2024/1689 as amended by Regulation (EU) 2026/1744 (Digital Omnibus on AI; OJ L, 2026/1744, 24 July 2026) [97].
